# Disautonomia: Uma Condição Esquecida – Parte II

**DOI:** 10.36660/abc.20200422

**Published:** 2021-05-06

**Authors:** Eduardo Arrais Rocha, Niraj Mehta, Maria Zildany Pinheiro Távora-Mehta, Camila Ferreira Roncari, Alan Alves de Lima Cidrão, Jorge Elias

**Affiliations:** 1 Universidade Federal do Ceará Faculdade de Medicina da UFC Hospital Universitário Walter Cantídio FortalezaCE Brasil Hospital Universitário Walter Cantídio da Universidade Federal do Ceará (UFC) - Programa de Pós-graduação em Ciências Cardiovasculares da Faculdade de Medicina da UFC, Fortaleza, CE - Brasil; 2 Universidade Federal do Paraná CuritibaPR Brasil Universidade Federal do Paraná, Curitiba, PR - Brasil; 3 Clínica de Eletrofisiologia do Paraná CuritibaPR Brasil Clínica de Eletrofisiologia do Paraná, Curitiba, PR - Brasil; 4 Universidade Federal do Ceará Faculdade de Medicina Departamento de Fisiologia e Farmacologia FortalezaCE Brasil Departamento de Fisiologia e Farmacologia - Faculdade de Medicina da Universidade Federal do Ceará (UFC), Fortaleza, CE - Brasil; 5 UFC Faculdade de Medicina FortalezaCE Brasil Programa de Pós-graduação em Ciências Cardiovasculares da Faculdade de Medicina da UFC, Fortaleza, CE - Brasil; 6 Vitória Apart Hospital Serviço de Eletrofisiologia VitóriaES Brasil Serviço de Eletrofisiologia do Vitória Apart Hospital, Vitória, ES - Brasil

**Keywords:** Disautonomias Primárias, Hipotensão Ortostática, Hipotensão Postural, Fadiga, Diabetes, Sistema Nervoso Autônomo, Síncope, neuropatia autonômica cardiovascular

## Manifestações clínicas gerais e cardiovasculares

As patologias que acometem o SNA podem manifestar-se de diversas formas, a depender da etiologia, do grau de comprometimento, do tempo de doença, da presença de comorbidades, da idade ou do uso de fármacos associados. Muitos sintomas podem ser completamente debilitantes, como as dores intensas na neuropatia periférica e as quedas ou síncopes nas neuropatias autonômicas. Pode ocorrer evolução para uma severa intolerância ortostática em casos mais avançados das disautonomias, na forma de hipotensão ortostática precoce, de dificil tratamento clinico, podendo estar associada a hipertensão supina, o que dificulta o tratamento (Tabela 1).^1^^–^^7^

**Tabela 1:** Sinais e sintomas clínicos e cardiovasculares de disautonomia e/ou neuropatia autonômica cardiovascular (NAC)

Sintomas clínicos	Sinais e sintomas cardiovasculares
Impotência masculina e redução da libido	Fadiga e intolerância ao exercício
Irregularidades na menorreia	Pré-síncope/síncope
Urgência e incontinência urinária	Escurecimento visual e intolerância a ortostase prolongada
Diarreia/Constipação/Empachamento	Quedas inexplicadas
Respostas exacerbadas a hipoglicemiantes	Respostas exacerbadas a anti-hipertensivos
Dificuldade de controle do diabetes (devido a gastroparesia)	Hipertensão supina
Hipo ou anidrose	Padrão *non-dipper* na MAPA
Alterações visuais, atrofia pupila	Cansaço, falta de ar (por incompetência cronotrópica)
Dor, dormência ou queimação em extremidades	Bradicardia
Esquecimento e redução da função cognitiva	Palidez, extremidades frias
Tremores, desequilíbrio	Hipotensão ortostática
Alterações no sono/Apneias	Síncope ou pré-síncope pós-prandial (até duas horas após refeição copiosa ou rica em carboidratos)
Dor forte na região cervical posterior (isquemia do trapézio)	Palpitações e taquicardia ao levantar

Fonte: próprio autor.

## Métodos de investigação

O Sistema nervoso autônomo (SNA) apresenta complexidade razoável, o que torna, à primeira vista, sua investigação difícil e de interpretação duvidosa. No entanto, alguns testes são simples e de fácil execução e fornecem informações valiosas quanto às suas deficiências. Eles podem ser realizados por meio de equipamentos computadorizados modernos ou de simples eletrocardiogramas digitais, que possam gravar os testes e os intervalos RR, tornando possíveis as medidas adequadas das relações entre as suas variações. Os objetivos desta avaliação são:

- Confirmar o diagnóstico;
- Estadiar a gravidade da disfunção;
- Identificar alterações subclínicas;
- Monitorar a evolução da doença.

Para a realização efetiva dos testes autonômicos, é essencial que o paciente esteja descansado e tranquilo. O laboratório de avaliação autonômica deve ser um local com pouco barulho, adequadamente aquecido e levemente escurecido.^8^^,^^9^

### Testes dos reflexos autonômicos cardiovasculares (testes de função cardiovagal)

**Figura 1:**
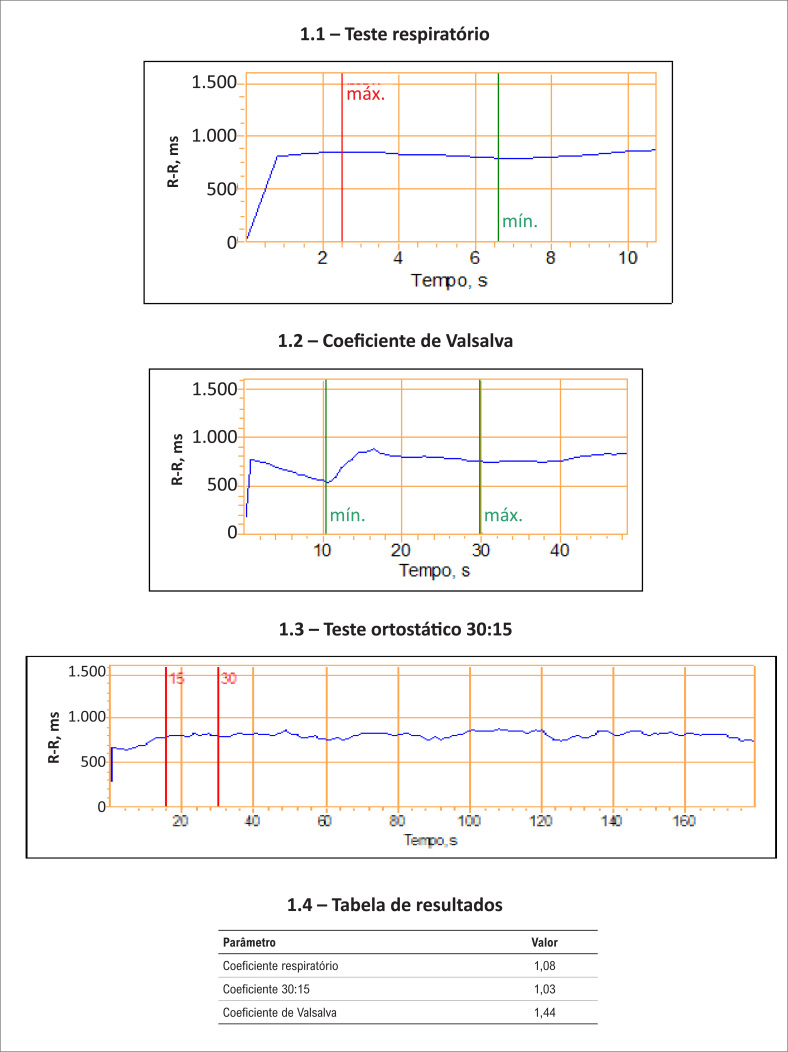
Testes dos reflexos autonômicos cardiovasculares. Caso clínico de paciente com disautonomia avançada e alterações nos testes respiratórios (1,08) e no coeficiente ortostático 30:15 (1,03). Os valores precisam ser ajustados para a idade e o sexo.^11^^–^^13^

Foram descritos por Ewing, na década de 1970, e são nos dias atuais os de padrão ouro no diagnóstico da NAC.^7^^,^^10^^-^^13^ Apresentam boa sensibilidade e especificidade, e devem ser realizados na presença de sintomas sugestivos de disautonomia, além de precocemente em pacientes que apresentem patologias como a diabetes, que podem evoluir com NAC mesmo na fase de intolerância à glicose (Figura 1).^8^^–^^10^ Os testes são divididos em métodos que avaliam as funções simpática e parassimpática, sendo alterados mais usual e precocemente, sobretudo no diabetes.

A presença de alteração em qualquer método dos três testes cardiovagais implica em neuropatia autonômica precoce ou incerta. O teste deve ser repetido após um ano, para confirmação e avaliação evolutiva. A presença de dois testes positivos mostra-se confirmatória para a NAC. A associação de hipotensão ortostática em dois testes positivos implica disautonomia avançada e com pior prognóstico.

Esses testes necessitam de uma avaliação e preparo adequado, com suspensão de diversos fármacos que possam alterar a análise da frequência cardíaca e do SNA. Pacientes com arritmias frequentes (mais de seis extrassístoles por minuto), fibrilação atrial, marca-passo cardíaco, tremores acentuados ou não colaborativos impossibilitam a avaliação adequada desse exame.

**Testes dos reflexos autonômicos cardiovasculares (testes da função cardiovagal)**

Descritos por Ewing na década de 1970, são os testes padrão-ouro no diagnóstico de neuropatia autonômica cardiovascular (NAC). Eles apresentam boa sensibilidade e especificidade e devem ser realizados quando houver sintomas sugestivos de disautonomia, além de precocemente em pacientes com patologias como diabetes, que podem evoluir com NAC, mesmo em sua fase de intolerância à glicose.^7^^,^^11^^-^^13^

Podem ser realizados por meio de equipamentos computadorizados modernos ou de simples eletrocardiogramas digitais, que gravem os testes, os intervalos RR. Isso possibilita as medidas adequadas das relações entre as variações dos maiores e menores intervalos RR.^14^^–^^16^

### Teste respiratório (quociente respiratório E/I)

Nesse método, verifica-se a relação (quociente) entre o maior ciclo RR na expiração, dividido pelo maior ciclo RR na inspiração: são feitos três ciclos de um minuto cada, com intervalos de um minuto entre os testes, o que possibilita a avaliação do sistema parassimpático.

Os ciclos de inspiração e expiração são lentos e profundos, com duração do ciclo respiratório total de dez segundos. O método acentua a arritmia respiratória sinusal, observada em indivíduos normais. A resposta normal é uma aceleração da frequência cardíaca durante a inspiração e uma desaceleração durante a expiração. Em suma, a frequência cardíaca é registrada no intervalo de um minuto (seis ciclos respiratórios lentos, profundos e com duração de dez segundos cada). A diferença entre a frequência cardíaca máxima e mínima, ou a razão desses dois valores (E:I) é registrada, sendo aferida em milissegundos.

Usualmente, calculamos a média das amplitudes respiratórias nos seis ciclos. Trata-se de um teste que avalia a resposta parassimpática a um estímulo respiratório. Pacientes com disautonomia podem apresentar oscilação reduzida ou ausente da frequência cardíaca à respiração profunda. A perda da arritmia sinusal respiratória pode ser um dos primeiros sinais de neuropatia autonômica diabética.

Os valores normais fisiológicos da diferença de amplitude são considerados quando os valores estão acima de 15 bpm. Entre 11-14 bpm, estariam classificados como limítrofe e abaixo de dez batimentos, patológico. A razão E:I (máxima frequência cardíaca, aferida em milissegundos, durante a expiração dividida pela máxima frequência cardíaca durante a inspiração) em indivíduos normais deve ser maior que 1,2.^11^^–^^15^ Tais valores devem ser ajustados de acordo com a idade e o sexo.

### Teste de Valsalva (quociente de Valsalva)

Neste teste, mede-se a relação entre o maior ciclo RR na fase de relaxamento, dividido pelo maior ciclo RR na fase da manobra de Valsalva. Isso permite a avaliação do sistema parassimpático predominante e também do simpático, quando associada às medidas de pressão arterial contínua.

A manobra de Valsalva é particularmente interessante, pois testa a integridade tanto da resposta parassimpática cardiovagal, através da análise da frequência cardíaca, como também da resposta simpática pelo estudo da pressão arterial. A técnica consiste basicamente em fazer o paciente, após estar monitorado, soprar durante 15 segundos por um pequeno tubo, com discreta saída de ar para evitar o fechamento da glote. A pressão expiratória de ar gerada deve ser em torno de 40 mmHg.

Ocorrem quatro fases distintas: as deflexões da pressão arterial nas fases I e III representam as perturbações mecânicas geradas por alterações da pressão intratorácica no início e final da manobra de Valsalva. Por outro lado, as fases II e IV são consideradas, de fato, as clinicamente relevantes.

Em indivíduos saudáveis, durante o esforço expiratório, há, na fase I, uma queda da pressão arterial pela diminuição do retorno venoso e que é percebida por barorreceptores intactos, que deflagram uma resposta que causa o aumento do tônus simpático, levando a vasoconstricção e ao aumento da frequência cardíaca.

Essa ação recupera a pressão na fase II tardia. Ao liberar a pressão intratorácica com o fim da manobra, há um retorno venoso aumentado e, com a manutenção ainda da vasoconstricção periférica, um *overshoot* (aumento exagerado) da pressão arterial (a combinação perfeita do aumento do retorno venoso e da vasoconstricção).

Pacientes com disfunção autonômica não conseguem reagir com aumento do tônus simpático à queda inicial da pressão provocada pela manobra. Assim, não há um aumento da pressão na fase II tardia e, tipicamente, não há o *overshoot* da pressão na fase IV. Ao invés disso, a resposta da pressão em pacientes com disautonomia revela um retorno gradual da pressão arterial a níveis basais, após a indução da hipotensão provocada pela fase expiratória forçada.^17^^–^^19^

Com relação à frequência cardíaca, o quociente de Valsalva é derivado pela máxima frequência cardíaca, medida em milissegundos, e gerada pela manobra de Valsalva, dividida pela menor frequência cardíaca nos primeiros 30 segundos do pico da frequência cardíaca. As respostas da frequência cardíaca são mediadas pelos barorreceptores.

O aumento da frequência cardíaca ocorre em decorrência da queda de pressão. Além disso, a resposta barorreceptora ao aumento da pressão excessiva da fase IV é a responsável pela bradicardia transitória ao final da manobra.^17^^–^^19^

Em indivíduos com disautonomia, detectam-se a perda do aumento excessivo da pressão e a ausência da bradicardia reflexa. Assim, a frequência cardíaca também não responde, devido à falta do aumento da resposta simpática, exibindo curva reta (ausência de oscilação da frequência cardíaca).

O quociente de Valsalva normal (máximo valor de RR em ms, dividido pelo menor valor de RR) durante a manobra deve ser maior que 1,21. Os valores limítrofes estariam entre 1,11 e 1,20, enquanto os valores patológicos seriam considerados quando menores ou iguais a 1,10.^13^^–^^15^ Essas aferições também devem ser ajustadas de acordo com a idade e o sexo.

### Teste do quociente 30:15 com a ortostase

Faz-se a avaliação do intervalo RR após a ortostase, em torno do 15º batimento (maior frequência habitual – menor intervalo RR) e do 30º batimento (menor frequência – maior intervalo RR), o que indica uma avaliação predominante do sistema parassimpático. O método mais simples e mais usado para testar o feedback cardiovascular é medir os parâmetros cardiovasculares (frequência cardíaca, pressão arterial e dosagem de noradrenalina) durante a mudança postural da posição horizontal para a ortostática.^16^^–^^19^

Ao ficar em pé, devido às mudanças na pressão hidrostática, 500-800 mL de volume são redistribuídos para os membros inferiores. Ao ficar em pé ativamente, no entanto, ocorre a compressão das veias dos membros inferiores (a chamada bomba musculoesquelética), aumentando imediatamente o retorno venoso.

Os mecanismos compensatórios agem rapidamente, fazendo com que a pressão arterial sofra poucas alterações em indivíduos saudáveis. No entanto, em 10 a 15% dos indivíduos, desordens circulatórias ortostáticas são observadas devido a insuficiência dos mecanismos compensatórios.

A avaliação da resposta à ortostase pode ser realizada por inclinação ativa ou pela resposta ao *tilt* *test*. Na primeira, no que diz respeito à frequência cardíaca, há um aumento rápido e máximo ao redor do décimo quinto batimento cardíaco em pessoas normais. Em seguida, há uma bradicardia relativa máxima ao redor do trigésimo batimento. Estudos farmacológicos indicam que esta resposta é mediada pelo nervo vago.

Pacientes com neuropatia autonômica cardiovascular diabética mostram apenas um leve aumento progressivo da frequência cardíaca.^19^ A razão 30:15 é usada como medida da integridade parassimpática. O intervalo RR mais longo se dá no 30º batimento. O intervalo RR mais curto ao 15º batimento é chamado de razão de Ewing ou razão 30:15, no qual um valor normal seria acima de 1,04.

Os *softwares* atuais não calculam mais a razão 30:15 pura; utilizam as medidas do intervalo RR mais longo entre o 20º e o 40º batimentos cardíacos e do intervalo RR mais curto entre o 5º e o 25º batimentos cardíacos.^14^^–^^19^

## Protocolo dos sete testes de avaliação de disautonomia

A associação dos três testes da função cardiovagal citados, com os testes de análise da variabilidade RR no domínio da frequência e a pesquisa de hipotensão ortostática) representa o protocolo dos sete testes (Figuras 1 e 2) para a pesquisa de NAC, com sensibilidade e especificidade elevada.

**Figura 2:**
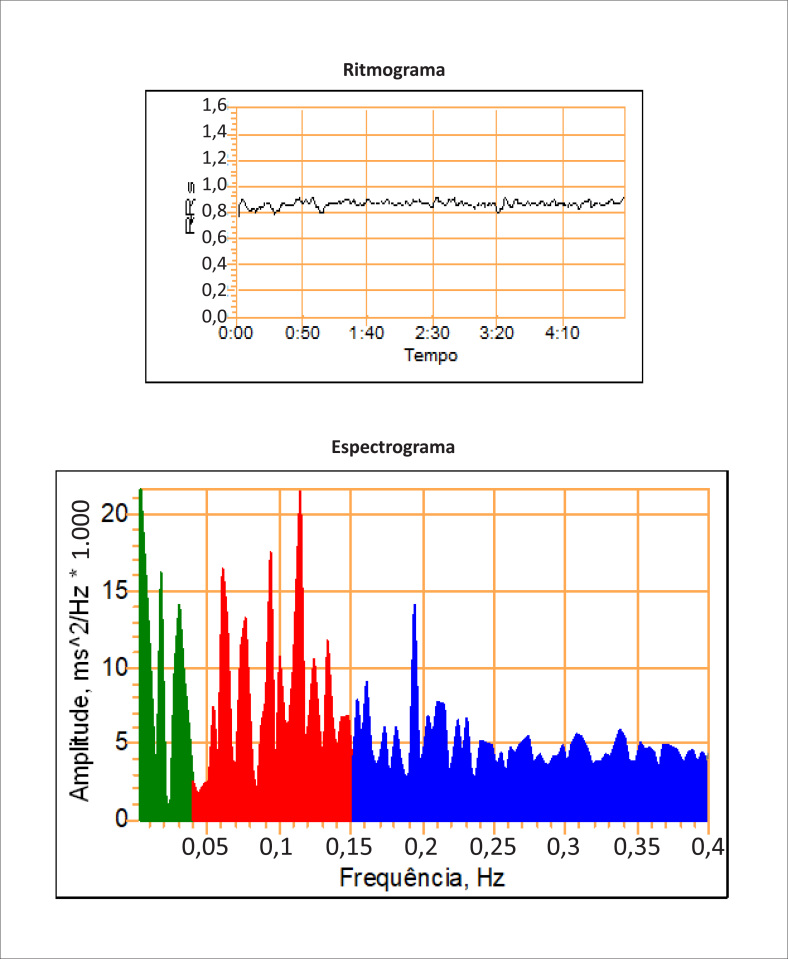
Análise da variabilidade RR no domínio da frequência durante ciclo de cinco minutos. Há necessidade de suspensão dos fármacos que possam interferir na análise da frequência cardíaca. A presença de arritmias frequentes, fibrilação atrial ou marca-passo impossibilita a análise do exame. **Componente de muito baixa frequência 0,01-0,04 Hz (FMB – VLF):** avaliação das flutuações do tônus vasomotor ligado a termorregulação e a sudorese (ação predominante do simpático). **Componente de baixa frequência 0,04-0,15 Hz (FB – LF):** avaliação do barorreceptor (componente simpático predominante com modulação vagal). **Componente de alta frequência 0,15-0,5 Hz (FA – HF):** relacionado ao controle sinusal (ação parassimpática). Pode ser realizado no protocolo e em conjunto com os testes da Figura 1 (configurando o protocolo dos sete testes de avaliação dos reflexos autonômicos cardiovasculares).

Ele pode apresentar melhor capacidade diagnóstica segundo alguns autores.^12^ Na presença de três métodos alterados dos sete testes é considerado positivo, e incerto ou precoce na presença de dois métodos alterados. A semelhança dos testes isolados cardiovagais, a presença de hipotensão ortostática associada inferem maior gravidade e severidade da NAC.

Os testes podem ser feitos em conjunto, através de *softwares* específicos como o da *Poly-Spectrum-Analysis*^®^, da *VNS-Rhythm*, ou *Neuro-Diag Ansar*^®^. Ambos são considerados de baixo custo quando comparamos aos equipamentos utilizados para tilt teste com medidas hemodinâmicas.

## *Tilt test* ou teste de inclinação ortostática

O *tilt* *test* é uma ferramenta diagnóstica muito útil em pacientes com disautonomia. Ao assumir a posição ortostática, há uma transferência de 500-800 mL de volume central para a periferia (pélvis, abdômen e membros inferiores), o que leva a uma queda no volume sistólico e, consequentemente, do débito cardíaco. Esse déficit, por sua vez, é sentido pelos barorreceptores do arco aórtico e do seio carotídeo, que após a interação com os centros vasomotores, deflagram uma resposta que gera uma redução da atividade parassimpática e o aumento da atividade simpática, que se traduz em vasoconstricção periférica e aumento da frequência cardíaca.

Este teste diagnóstico pode ser particularmente útil para detectar e confirmar a falência autonômica observada na hipotensão ortostática, na taquicardia postural ortostática, na hipotensão ortostática tardia e, obviamente, nas alterações reflexas da reação vasovagal.^19^^–^^20^

O *tilt* *test* é realizado em uma sala com pouco barulho e distrações mínimas. O paciente é incialmente instruído a ficar em jejum por três horas e deitar em posição supina, pelo período mínimo de 10 a 15 minutos. Embora existam vários protocolos, a recomendação atual (e o protocolo mais utilizado) são a inclinação a 70 graus por cerca de 30 a 40 minutos.^21^

O teste provocativo com isoproterenol ou 1,25 mg de isossorbida sublingual pode ser útil para investigar a síncope de origem vasovagal por aumentar a sensibilidade do teste.^22^ No entanto, a sensibilização com fármacos não se aplica quando o objetivo é avaliar a disautonomia, pois convém avaliar a resposta fisiológica cardiovascular espontânea ao estresse ortostático prolongado.

Embora as medidas intermitentes da pressão arterial (a cada 2-3 minutos) possam ser adotadas, a monitorização contínua da pressão arterial e do registro eletrocardiográfico seria preferível, ainda que com o custo bem mais elevado.

## *Tilt test* associado às medidas hemodinâmicas

A utilização de módulos adicionais à medida da pressão arterial contínua (*modelflow* do *Finapress*^®^ e cardiografia por impedância do *Task Force Monitor*^®^) permite determinar de modo indireto o volume sistólico. A partir desse ponto, os parâmetros de caráter hemodinâmico podem ser estimados com razoável precisão, tais como a resistência vascular periférica e o débito cardíaco.

O *modelflow* do *Finapress*^®^ utiliza a análise do contorno do pulso arterial, uma técnica capaz de determinar o volume sistólico. No *modelflow*, a onda de fluxo do pulso arterial é calculada a partir do contorno da pressão de pulso arterial, cuja integração a cada batimento cardíaco gera o volume sistólico.^22^^,^^23^

Por outro lado, a cardiografia por impedância (*Task Force Monitor*^®^) mede as alterações na impedância torácica gerada por volumes sanguíneos flutuantes durante o ciclo cardíaco, permitindo o cálculo de volume sistólico, do débito cardíaco e de outros parâmetros.^23^ Essas técnicas não invasivas utilizadas para determinar o débito cardíaco, ainda que não totalmente precisas, foram validadas quando comparadas às técnicas invasivas e se mostram bastante confiáveis em acompanhar as mudanças relativas do débito cardíaco.

Portanto, o *tilt test* com parâmetros hemodinâmicos e a aferição da pressão arterial contínua permitem avaliar o volume sistólico, o débito cardíaco e a resistência vascular periférica. A análise dos parâmetros hemodinâmicos durante o *tilt test* é muito importante, pois permite registrar a queda na resistência vascular periférica, revelando a presença de disautonomia, muitas vezes sem a queda significativa na pressão arterial, devido a mecanismos compensatórios limítrofes (disautonomia leve a moderada). Além disso, possibilita determinar se há importante redução do volume sistólico, que pode ser um componente não neurogênico da hipotensão ortostática (por desidratação crônica, por exemplo).

Dessa forma, o *tilt test* com parâmetros hemodinâmicos permite identificar alterações subclínicas da integridade do SNA, mesmo sem a evidente queda de pressão, o que aumenta a sensibilidade do método. A limitação do método é seu elevado custo, sendo restrito a poucos centros, principalmente de pesquisas em disautonomia.

## MAPA 24 horas

Os balanços autonômicos diurno e noturno não afetam apenas a frequência cardíaca, mas também a pressão arterial. Normalmente, a pressão arterial flutua, com níveis mais elevados durante a vigília e queda à noite (descenso noturno). Quedas proporcionais da pressão arterial à noite, tendo como referência o período diurno, determinam as respostas de descenso noturno esperado: resposta atenuada (0 a 10% de descenso noturno), resposta normal (10-20% de descenso noturno), resposta acentuada (acima de 20% de descenso) e resposta reversa (elevação da pressão arterial ao invés de descenso noturno esperado).

A resposta atenuada ou reversa demonstra atividade simpática exacerbada, podendo estar presente na disautonomia, e tem sido associada ao aumento de mortalidade. Além disso, a presença de hipertensão noturna pode aumentar o risco de hipotensão diurna (resultado, entre outros aspectos, do incremento da excreção noturna de hormônio natriurético).

A MAPA pode indicar alterações relacionadas a disfunção autonômica cardiovascular, podendo selecionar pacientes para avaliação mais profunda de disautonomia. Mais especificamente, a MAPA pode ser bastante útil em detectar hipertensão noturna (um importante preditor de eventos cardiovasculares) e formas de hipotensão ortostática precoce ou pós-prandial, habitualmente não descobertos com as medidas usuais de pressão arterial.

**Hipertensão supina - Um sinal de Alerta!**

A presença de hipertensão supina, padrão non-dipper ou negativo dipper no exame da MAPA 24 horas, principalmente em pacientes acometidos de patologias conhecidas que podem evoluir com disautonomia deve levar a suspeição e investigação com screening clínico e laboratorial para Disautonomia.

## *Holter* e análises da variabilidade RR

O nó sinusal está sujeito tanto a ação simpática quanto a parassimpática (vagal), dependendo da modulação avaliada. Posição em pé, estresse mental e exercício estão associados a um aumento do tônus simpático. O tônus vagal, por outro lado, é aumentado em condições de repouso. Em indivíduos normais, tanto o tônus simpático quanto o parassimpático flutua ao longo do dia, gerando uma variação nos intervalos RR, ou simplesmente, variabilidade RR, que, em indivíduos normais, declina de três a cinco batimentos por década.

O Holter 24 horas pode ser utilizado para a análise da frequência cardíaca média, incompetência cronotrópica e arritmias cardíacas, além de, quando acoplado a softwares específicos, permitir a análise da variabilidade RR.

A análise da frequência cardíaca média pode sugerir disfunção autonômica no diabético, indicar uma taquicardia sinusal inapropriada (TSI) ou permitir a identificação de incompetência cronotrópica. A detecção de arritmias pode sugerir outras etiologias como justificativa dos sintomas, além de auxiliar na seleção para a realização dos testes cardiovagais.

Existem vários métodos de estudo dos dados da variabilidade RR, incluindo a análise no domínio do tempo e da frequência. Na análise no domínio do tempo, cada QRS é detectado para determinar o intervalo “normal a normal” que, por sua vez, fornece informação adicional, incluindo o seu desvio-padrão. A análise estatística mais complexa requer períodos prolongados de tempo. A análise espectral pode fornecer avaliação no domínio da frequência e informar como a variância é disposta, como uma função da frequência.

As alterações na frequência cardíaca ocorrem continuamente durante as atividades diárias, refletindo no equilíbrio autonômico, nos mecanismos cardiovasculares reflexos e nos estímulos externos. Em pessoas normais, a variabilidade RR aumentada da frequência cardíaca é considerada uma medida de integridade autonômica, enquanto a variação reduzida da frequência cardíaca é um sinal precoce de desequilíbrio autonômico.

A análise da variabilidade RR pode ser feita no domínio do tempo e da frequência, durante períodos curtos de poucos minutos ou em intervalos mais longos (*Holter* de 24 horas). A análise no domínio do tempo inclui a avaliação de vários parâmetros, como: média dos intervalos normais, média da frequência cardíaca, diferença da frequência cardíaca máxima, desvio-padrão na média de cinco minutos dos intervalos RR normais (SDANN) e raiz quadrada da média da diferença dos intervalos RR sucessivos (rMSSD).

Monitorizações prolongadas (Holter 24 horas) permitem calcular, também, o número das instâncias por hora em que foi medida uma diferença maior que 50 ms, entre dois intervalos RR consecutivos (pNN50). Essencialmente, todos esses índices exploram a atividade parassimpática.

A análise espectral da variabilidade RR (domínio da frequência), por sua vez, revela três componentes de frequências principais:

1. Componente de frequências muito baixas (< 0,04Hz), relacionado com flutuações do tônus vasomotor e ligadas a termorregulação e a sudorese (controle simpático);
2. Componente de frequências baixas (0,04-0,15 Hz), ligado ao reflexo barorreceptor (controle simpático com modulação vagal);
3. Componente de frequências altas (0,15-0,4 Hz), influenciado pela respiração (arritmia sinusal respiratória) e sendo componente de atividade parassimpática.

Em pacientes diabéticos e com disfunção predominantemente vagal (mais precoce), a amplitude de frequências altas está reduzida ou ausente. Por outro lado, nas disfunções simpáticas mais tardias, as amplitudes de frequências baixas e muito baixas estão reduzidas.

Os parâmetros do domínio do tempo, o poder total espectral da variabilidade RR e o componente espectral de frequências altas são parassimpáticos. Embora o componente de frequência baixa seja controlado pelo simpático, a ativação extrema simpática (como no exercício, na insuficiência cardíaca) atenua a variabilidade RR, dificultando seu registro, podendo, então, não se correlacionar com a atividade simpática real.

Dessa maneira, atualmente é aceito que o “poder espectral” absoluto das frequências baixas não reflete a atividade simpática. No entanto, quando medida em termos relativos (como uma percentagem da variabilidade RR global), a proporção da baixa frequência sobre as altas frequências providencia uma indicação média e aproximada da modulação simpática do coração.

Portanto, a razão do componente de baixa frequência em relação à alta frequência é um parâmetro mais representativo de status simpático. Como a variabilidade RR é influenciada pela idade, pelo sexo e pelo ritmo respiratório, recomenda-se o ajuste para essas variáveis. Os resultados da análise espectral correlacionam bem com os testes de função autonômica nas situações clínicas.

A análise espectral é mais sensível em estágios iniciais da NAC. Nos pacientes diabéticos, em especial, uma deterioração progressiva dos parâmetros da análise espectral relacionada ao sistema parassimpático é documentada. O esperado aumento noturno da banda de alta frequência da variabilidade RR, que representa a modulação vagal do coração, parece ser a anormalidade mais precoce detectada. Durante os estágios avançados da NAC, todos os componentes são eliminados.

## Eletromiografia e as neuropatias de fibras finas

As fibras autonômicas pós-ganglionares são do tipo C, não-mielínicas. Junto às fibras Aδ, pouco mielinizadas, compõem o grupo das fibras finas. Diferenciam-se das fibras mielinizadas grossas por sua espessura e pela velocidade: conduzem impulsos nervosos numa velocidade que varia de 0,5 a 1 m/s, enquanto nas últimas se observam velocidades de até 120 m/s.^24^^,^^25^

As fibras Aδ são do tipo sensoriais somáticas e participam da inervação cutânea, mediando a percepção de estímulos álgicos e térmicos. As fibras autonômicas tipo C inervam a musculatura cardíaca, os músculos lisos (presentes na parede dos vasos sanguíneos, tratos gastrintestinal e geniturinário) e as glândulas (salivares, lacrimais e sudoríparas).^24^

O comprometimento neuropático de fibras finas pode ocorrer sem que aconteça o mesmo às fibras grossas, o que configura a neuropatia de fibras finas, ou no contexto de uma polineuropatia, onde há um acometimento claro. Nas neuropatias de fibras finas, mais comumente observa-se o acometimento das fibras Aδ.

Entre os sintomas típicos, destacam-se parestesia, dor e sensação de queimação ou de frio, com clara piora no período noturno. Sintomas e sinais disautonômicos ocorrem em aproximadamente 50% desses pacientes. Mais raramente, um quadro de neuropatia de fibras finas pode manifestar-se predominante, com sintomas autonômicos.^26^^,^^27^

A biopsia de pele e o teste de quantificação sensitiva (QST – *Quantitative Sensory Testing*) são úteis principalmente na avaliação das fibras Aδ, ainda que existam outros exames direcionados à avaliação das fibras autonômicas que, quando realizados, aumentam a sensibilidade diagnóstica.^26^^–^^31^.

A eletroneuromiografia convencional, através de estudos de condução e eletromiografia, é um exame fundamental na avaliação inicial desses casos, não para confirmar o diagnóstico, mas, sobretudo, para excluir polineuropatia (acometimento de fibras grossas) e investigar condições que possam manifestar-se de modo semelhante aos quadros de neuropatia de fibras finas, como a radiculopatia S1 bilateral que, caracteristicamente, cursa com parestesias nos pés.

Os estudos de condução por esse método avaliam apenas as fibras nervosas mais rápidas, sendo incapazes de identificar o comprometimento das fibras finas. Portanto, nos quadros puros de neuropatia de fibras finas, os estudos de condução, incluindo a avaliação dos nervos surais, que classicamente estão alterados nos quadros de polineuropatias, serão normais.^26^^,^^27^^,^^31^^,^^32^

A biopsia de pele ainda é considerada o padrão-ouro no diagnóstico de neuropatia de fibras finas. É um procedimento pouco invasivo, realizado ambulatorialmente e com anestesia local. Em geral, retira-se um fragmento de 3 mm de tecido da região distal de um dos membros inferiores, 7 a 10 cm proximal ao maléolo lateral. Outros fragmentos podem ser retirados, 7 a 10 cm proximal ao joelho e distal ao trocânter maior, do mesmo membro, respectivamente, para definir o padrão – comprimento-dependente (padrão distal para proximal), ou não comprimento-dependente, ou a biopsia de sítios específicos diante de sintomas focais.

Conforme já mencionado, a avaliação da densidade de fibras intraepidérmicas é direcionada predominantemente às fibras Aδ.^28^ Os valores normais esperados variam de acordo com a idade, o gênero e o sítio de biopsia, e estudos recentes procuram normatizá-los.^33^^,^^34^ Quando os valores são desconhecidos para um determinado sítio, a análise comparativa com o lado contralateral pode ser uma alternativa válida.

As limitações do método são: dificuldade de acesso a laboratórios especializados, principalmente no contexto de países emergentes como o Brasil; a possibilidade de o exame ainda ser normal no início do quadro; a ausência de normatização de valores esperados para diversos sítios anatômicos e a falta de padronização para avaliar as fibras autonômicas.

## Teste sensorial quantitativo e teste sudomotor

O teste QST (*Quantitative Sensory Testing*) avalia o limiar de sensação da dor, sobretudo, através de estímulo térmico controlado pelo calor, de modo geral. Avalia, então, a integridade das fibras Aδ. Trata-se, portanto, de um exame que depende da colaboração do paciente, uma vez que ele precisa sinalizar as suas percepções adequadamente. Dessa forma, ele pode ser falseado pela dificuldade de compreensão das instruções, do paciente concentrar-se ou mesmo por ação volitiva.

Outras limitações ao método são a baixa disponibilidade e o fato de que ele não distingue lesões periféricas das centrais. O comprometimento das vias espinotalâmicas e de áreas cerebrais relacionadas a esta modalidade sensitiva também levarão a um padrão de anormalidade no exame. Por esses motivos, não é recomendado que o método seja utilizado como teste único na definição diagnóstica de neuropatia de fibras finas.^26^^,^^30^

Os exames das fibras autonômicas são direcionados principalmente à avaliação da função sudomotora. A produção de suor pelas glândulas sudoríparas é mediada pela inervação simpática colinérgica. Observa-se, de modo geral e diante de um comprometimento de tais fibras em padrão comprimento-dependente (distal para proximal), anidrose em distribuição de botas e luvas, com hiperidrose proximal compensatória.^26^^,^^27^ A perda grave e difusa dessa função pode levar a distúrbios da termorregulação e hipertermia.

Dos métodos disponíveis, pode-se ser feito o teste do suor termorregulador (*Thermoregulatory Sweat Testing – TST*) e o teste quantitativo do reflexo axonal sudomotor (*Quantitative Sudomotor Axon Reflex Testing – QSART*).

Realiza-se o *TST* em uma sala onde é possível controlar a temperatura e a umidade. O paciente deita em posição supina em uma maca, com sua temperatura sendo monitorada por dois sensores (um para a pele e outro para a cavidade oral) e o corpo coberto por um composto, que muda de cor diante da alteração de pH local, produzido pelo suor.

A temperatura da sala é elevada para 45-50 °C, mantendo-se uma umidade relativa do ar em torno de 35-40%. A temperatura da pele é mantida entre 38,5 e 39,5 °C, e a temperatura oral deve elevar-se em 1 °C em relação ao valor basal ou alcançar 38 °C (o que for maior). A observação deve ocorrer em um período de tempo entre 30 e 65 minutos. A mudança de cor do reagente sobre o corpo do paciente indica a produção local de suor. Então, são registradas fotografias digitais e gerado um mapa anatômico da densidade de suor, que será posteriormente interpretado.

As principais limitações do método são a pouca disponibilidade e a sua incapacidade de distinguir comprometimento pré ou pós-ganglionar. Desse modo, a combinação com um exame direcionado às fibras pós-ganglionares pode ajudar a dirimir eventuais dúvidas.^31^^,^^34^

O QSART avalia a função das fibras autonômicas pós-ganglionares relacionadas à função sudomotora e a produção de suor através do estímulo colinérgico realizado por técnica de iontoforese. Habitualmente, são avaliados quatro segmentos – antebraço, perna proximal, perna distal e dorso do pé; isso pode fornecer informações sobre o padrão de acometimento: comprimento-dependente ou não comprimento-dependente.

O sistema é composto por uma cápsula especial multicompartimentalizada (uma subdivisão para o estímulo iontoforético, outra para mensurar a umidade e uma terceira que separa as duas primeiras), que fica em contato direto com a pele; e um sistema de fluxo contínuo de nitrogênio seco, que passa pela cápsula à temperatura constante e segue para um higrômetro, onde se registra a oscilação de umidade frente à produção local de suor.

Registra-se a variação de umidade em forma de gráfico, em um computador acoplado ao sistema. Este gráfico é analisado principalmente quanto à sua latência e à área sobre a curva, com valores padronizados para homens e mulheres. As limitações do método são a dificuldade de acesso e a impossibilidade de avaliar fibras pré-ganglionares.^26^

## Ressonância magnética cerebral e cintilografia com MIBG

A ressonância magnética pode ser útil no diagnóstico de atrofia multissistêmica (AMS), ao identificar alterações estruturais específicas no cérebro, focadas na localização de padrões de atrofia da substância cinzenta.^35^^,^^36^

A visualização de imagens ponderadas em T1 e T2 por neuroradiologistas experientes tem identificado sinais clássicos como *o da cruz* (que representa a degeneração de fibras da ponte e pontocerebelares, com preservação do trato córtico-espinal). Este sinal aparece como uma cruz hiperdensa na ponte, com alta especificidade (97%), embora com baixa sensibilidade (50%). Outra observação é o sinal hiperdenso na borda do putâmen com alta especificidade (90%), embora também com menor sensibilidade (72%).^35^^,^^36^

Nos últimos anos, houve um progresso significativo nas técnicas de neuroimagem, através do uso de novas conectividades e técnicas funcionais, que podem melhorar a acurácia diagnóstica e determinar novos marcadores de progressão da doença. Uma abordagem multimodal com tecnologias inovadoras como parte do arsenal diagnóstico, permitirá futuros progressos nesta área e na pesquisa da atrofia multissistêmica (AMS).^35^^,^^36^ Com relação à doença de Parkinson, a análise morfológica do mesencéfalo pela ressonância magnética, sobretudo da substância negra e dos núcleos da base, apresenta achados que auxiliam no diagnóstico das síndromes parkinsonianas.^36^

Uma nova e excitante área na ressonância magnética é a análise da inflamação cerebral. Em pacientes com síndrome da fadiga crônica, a inflamação cerebral tem sido investigada com espectroscopia, através da medição dos níveis de vários metabólitos relacionados à neuroinflamação, incluindo os compostos que contém colina, mio-inositol, lactato e N-acetilaspartato.^37^ Um estudo que avaliou a espectroscopia com ressonância magnética, aplicada à área cerebral inteira, demonstrou anormalidades de metabólitos e temperatura de forma distribuída em todo o cérebro, ao invés de regionalmente limitada.^37^

O achado sugere que a síndrome da fadiga crônica seja um processo patológico difuso que afeta todo o cérebro, o que é consistente com os seus sintomas clínicos heterogêneos. Estes estudos, segundo os autores, sustentam a hipótese de que a síndrome da fadiga crônica seja resultado de uma neuroinflamação crônica e de baixa intensidade.

Outro dado interessante gerado pela ressonância magnética é a análise dos distúrbios cognitivos nas alfassinucleinopatias.^38^ Muito desses pacientes pacientes apresentam hipotensão ortostática, o que leva à hipoperfusão cerebral transitória. Uma hipótese sugerida é que a hipoperfusão cerebral (transitória ou repetitiva) pode ser a responsável pelo déficit cognitivo detectado nesses pacientes.

A ressonância magnética estrutural demonstra hiperintensidade da substância branca, o que pode contribuir para defeitos cognitivos. Há evidências que a HO está associada a hiperintensidade de substância branca nas alfassinucleinopatias, explicando parcialmente a relação entre hipotensão ortostática e deficiência cognitiva.

Novas aplicações de ressonância magnética funcional mostram que flutuações fisiológicas na substancia branca observadas na ressonância, precedem as alterações estruturais da hiperintensidade da substância branca, sendo medidas mais sensíveis para avaliar o comprometimento cerebral.^38^

As imagens de cintilografia com metaiodobenzilguanidina (MIBG) podem ser utilizadas para quantificar diretamente a inervação simpática cardíaca em várias patologias, inclusive as neuropatias autonômicas cardiovasculares. A assimetria de inervação pode ser responsável por predisposição a arritmias e morte súbita, e também ser utilizada para avaliar a reinervação simpática após o tratamento adequado.^39^

## Testes laboratoriais

As catecolaminas plasmáticas mais importantes em humanos são a epinefrina e norepinefrina, ambas refletem a atividade simpática. A norepinefrina é liberada em terminais simpáticos neuronais; apenas uma pequena taxa chega até a circulação sistêmica. A epinefrina, por sua vez, é liberada por meio da estimulação pré-ganglionar simpática da medula adrenal. A epinefrina e a norepinefrina plasmáticas respondem de modo diverso aos agentes estressores. Enquanto a norepinefrina responde mais aos estímulos do frio, a epinefrina apresenta uma maior resposta à hipoglicemia e às hipotensões.^40^

Ficar em pé após o repouso na posição deitada, ou inclinar o paciente no *tilt test*, traz como consequência o acúmulo de sangue nos membros inferiores e resulta em queda do débito cardíaco. A ativação reflexa do sistema nervoso simpático resulta, entre outras ações, no aumento na liberação de norepinefrina pelos terminais dos nervos simpáticos, o que resulta no aumento de até 100% na circulação plasmática de norepinefrina em um período de cinco minutos.

Pacientes com falência autonômica secundária à disfunção dos neurônios pós-gangliônicos simpáticos podem ter concentrações reduzidas de norepinefrina em posição supina. Por outro lado, indivíduos com falência autonômica por qualquer causa frequentemente falham em elevar seus níveis de norepinefrina plasmática ao ficarem em pé ou serem inclinados no tilt test.

Isso ocorre devido à redução ou a ausência de disparos dos eferentes simpáticos em resposta ao estímulo ortostático. Um incremento subnormal de norepinefrina ao estresse ortostático é um dado bastante específico, embora não muito sensível, da resposta simpática atenuada pela falência barorreflexa-simpatoneural ou pela denervação simpática.^40^

Por outro lado, em pacientes com disfunção autonômica hiperadrenérgica, tais como os que apresentam prolapso de valva mitral ou alguns subtipos da síndrome postural ortostática taquicardizante (SPOT ou POTS na língua inglesa), pode haver incremento supranormal exagerado de norepinefrina, quando submetidos ao estresse ortostático (ficar de pé ou inclinados).

Na hipotensão ortostática neurogênica, causada por diversas desordens autonômicas, incluindo a neuropatia autonômica cardiovascular (NAC), o incremento ortostático de norepinefrina é atenuado. Portanto, um incremento de norepinefrina plasmática menor que 60% após cinco minutos de ortostase favorece o diagnóstico de hipotensão ortostática neurogênica.^40^

Outros exames laboratoriais específicos podem ser solicitados a fim de investigar as diversas etiologias potencialmente causadoras de disautonomia conforme a sintomatologia e a suspeita clínica. Patologias como diabetes, amiloidose, insuficiência renal, doenças autoimunes e neoplasias, sobretudo de pulmão, podem necessitar de investigação especializada.

**Neuropatia periférica = sinal de alerta**

A presença de sintomas ou do diagnóstico de neuropatia periférica pode representar um sinal de alerta para a pesquisa de disautonomia. Nos pacientes diabéticos, mais de 50% apresentarão neuropatia autonômica cardiovascular (NAC) quando diagnosticados com neuropatia periférica, enquanto quase 100% dos pacientes com NAC terão neuropatia periférica.

## Tratamento

**Figura 3:**
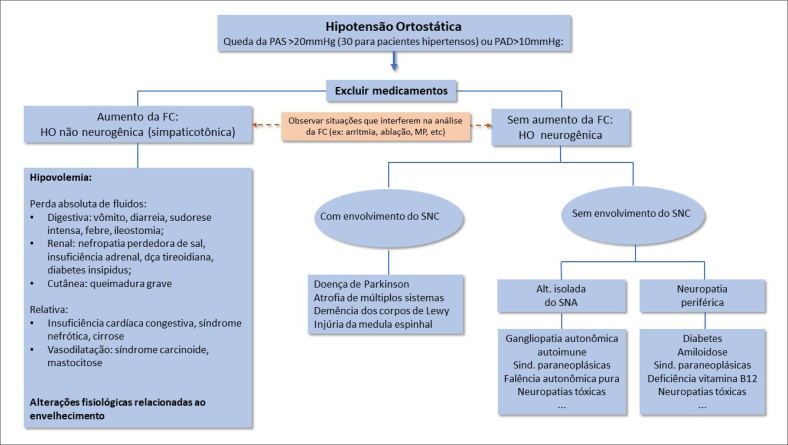
Fluxograma de abordagem da hipotensão ortostática e seus diagnósticos diferenciais. Dividido em grupos com aumento da frequência cardíaca em ortostase, usualmente observada em HO por hipovolemia ou medicamentosa e sem aumento, visto nas hipotensões neurogênicas e excluindo-se o uso de fármacos bradicardizantes ou pacientes com doença do nó sinusal.

Na maioria dos casos, o tratamento da disautonomia e, particularmente, da hipotensão ortostática (HO), bem como sua principal manifestação clínica, devem seguir uma abordagem progressiva, que passa tanto pelo tratamento não farmacológico como pelo uso de fármacos (Figuras 3 e 4).^41^^–^^44^

**Figura 4:**
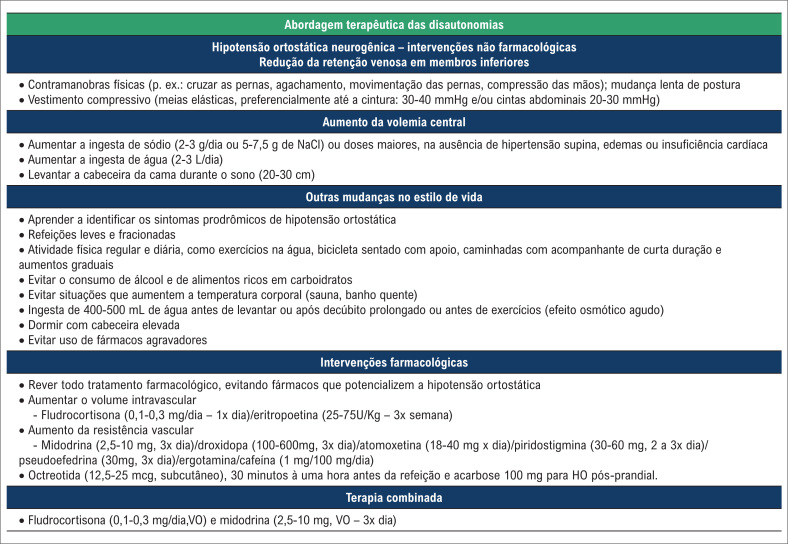
Abordagem terapêutica das disautonomias.

Fonte: próprio autor.

O objetivo do tratamento dos pacientes com HO é a melhora dos sintomas clínicos debilitantes (especialmente a redução do risco de quedas) e da qualidade de vida, aumentando a tolerância a maiores períodos de ortostase e a capacidade física. A busca por níveis de pressão arterial normais dificilmente é atingível.

A necessidade de tratamento deve estar baseada em uma análise individualizada, tomando como referência a gravidade da apresentação clínica e as comorbidades envolvidas. Em boa parte dos casos, particularmente nos pacientes idosos e/ou com disautonomia, deve-se buscar um melhor controle dos sintomas e dos sinais vitais em ortostase, visando ajudar na otimização da terapêutica instituída.

Existe uma carência de estudos sobre o tratamento da HO, sendo que as recomendações existentes estão baseadas, sobretudo, em pequenos trabalhos. Uma limitação potencial é o fato de não terem sido validados por estudos randomizados, com um número mais significativo de pacientes. Além disso, eles refletem apenas o resultado do tratamento agudo da HO e, de modo geral, em um grupo heterogêneo de pacientes. Trata-se de um aspecto fundamental, já que a HO envolve diversas patologias que se distinguem na sua forma de apresentação e evolução clínica.^42^^–^^44^ Um consenso de especialistas em HO neurogênica (HON) propôs um tratamento escalonado e baseado em quatro etapas:

1. Avaliar e ajustar os medicamentos preexistentes;
2. Tratamento não farmacológico;
3. Implementar a monoterapia;
4. Tentar a associação de fármacos.

Segundo esses autores, existe uma recomendação de que para cada etapa proposta do tratamento exista um período mínimo de duas semanas de observação, a fim de definir o benefício sintomático antes da migração para outra estratégia.^45^^,^^46^

Os envolvidos no tratamento dos pacientes com disautonomia devem sempre lembrar que a educação do paciente, dos familiares e dos cuidadores sobre os mecanismos envolvidos na gênese da HO e as situações da atividade diária que podem favorecer uma queda da pressão arterial são fundamentais no tratamento clínico. Exemplos como o de permanecer em ambientes com temperatura elevada, banhos quentes, tipo e intensidade de esforço físico, postura ortostática prolongada ou atingida de forma rápida, ingestão de bebida alcoólica ou de grandes refeições, particularmente com carboidratos, podem precipitar ou agravar os sintomas.

## Tratamento não farmacológico

### Análise dos medicamentos em uso

Independente da etiologia da disautonomia, sempre que possível deve ser considerada a interrupção do uso ou o ajuste posológico dos medicamentos que potencializam a HO.^42^^,^^43^^,^^47^ Um número substancial desses agentes são medicamentos de uso regular por parte dos cardiologistas.

Na medida em que se faz o ajuste dos medicamentos, é importante manter um monitoramento constante dos sintomas de HON. Para que isso aconteça, alguns estudos recomendam a utilização de questionários criados com este fim.^43^^,^^44^^,^^47^ Nos casos com indicação definida, deve-se optar por anti-hipertensivos com uma meia-vida menor, preferencialmente com dosagem noturna única.

Medicamentos como os nitratos e diuréticos, que diminuem a pré-carga, devem ser suspensos ou evitados. Outros fármacos que também pioram ou contribuem para a HO são aos fármacos dopaminérgicos, anticolinérgicas, antidepressivos tricíclicos, alfa-1-bloqueadores (p. ex., tansulosina) e outros anti-hipertensivos.

### Medidas não farmacológicas

A próxima etapa do tratamento é a incorporação de várias medidas não-farmacológicas na rotina diária do paciente, todas com o objetivo de minimizar os sintomas decorrentes da HON. Do ponto de vista prático, elas são incorporadas ao mesmo tempo em que se faz uma revisão cuidadosa do tratamento farmacológico previamente ministrado.

No caso de pacientes que apresentem sincope, pré-síncope ou quedas recorrentes, existe uma urgência maior em eliminar a instabilidade postural causada pela HO e orientar os pacientes sobre as manobras que possam reduzir a retenção venosa nos membros inferiores e no trato digestório.^48^

As medidas não farmacológicas podem ser usadas individualmente; no entanto, são mais eficazes quando usadas em combinação ou durante a titulação concomitante de tratamentos farmacológicos. Embora sejam custo-efetivas e passíveis de combinação com as intervenções farmacológicas, as orientações não farmacológicas podem apresentar baixa aderência por parte dos pacientes.

### Aumento do volume circulatório

Pacientes com HON precisam de intervenções que possibilitem a normalização ou a expansão do volume sanguíneo. Muitos deles, especialmente os idosos, apresentam uma diminuição volêmica secundária a uma ingesta inadequada de líquido por via oral, o que pode ser decorrente de uma restrição voluntária para evitar condições comuns, como a urgência urinária do idoso ou de pacientes com doenças neurológicas.^49^

O ajuste do volume de ingesta de fluidos também deve levar em consideração a área geográfica e as oscilações climáticas. A ingesta de água é considerada o “fármaco” de primeira escolha no tratamento da HON.^49^^–^^53^

Além disso, em situações agudas (p. ex.: síncope ou HO muito sintomática), quando existe programação de longo período de ortostase ou exposição ao calor, recomenda-se o uso de ingesta rápida de água, preferencialmente gelada (500 mL ingerida no período de 2 a 3 minutos), devido à promoção do aumento do tônus simpático e à consequente elevação da PA.^53^^–^^56^

A resposta pressórica aguda inicia-se depois de cinco a dez minutos após a ingesta de água, com pico entre 20 e 40 minutos, ou seja, produz um efeito que mimetiza o uso de fármacos de efeito rápido e curta duração. O efeito dessa ingesta rápida de água se deve ao reflexo hipo-osmolar na circulação portal e pode durar até uma hora, possibilitando uma melhora dos sintomas de HON. A ingesta de outros líquidos é ineficaz para gerar uma resposta pressórica significativa. Dessa forma, a hidratação adequada pode produzir efeitos agudos e crônicos, com impacto clínico benéfico nos pacientes com HON.^51^^–^^56^

### Ingesta de sódio

Outro tratamento não farmacológico importante é o monitoramento e o ajuste do aporte diário de sal. Como o sódio é considerado um componente negativo da dieta, muitos pacientes buscam remover ou reduzir significativamente o seu uso na dieta, piorando os sintomas ortostáticos.

Recomenda-se aos pacientes com HON uma ingesta de 2 a 3 de sódio ao dia (5 a 7,5 g de sal). Alguns casos podem necessitar de aportes maiores, chegando até 10 g de sódio. Pacientes com risco de insuficiência cardíaca, hipertensão supina ou edema periférico devem ser monitorados de perto, pela possibilidade de piora dos sintomas, já que podem necessitar ajustes ou menores ingestas. Deve-se evitar a privação acentuada de sal.^57^^,^^58^

### Dieta

Em pacientes com HO, a ativação simpática não é capaz de compensar o acúmulo de sangue na circulação esplâncnica após a refeição. A atividade simpática vasoconstritora na HON é deficiente e muitos pacientes apresentam hipotensão significativa após a ingesta de alimentos.

Para os indivíduos com hipotensão pós-prandial, recomenda-se refeições em menor quantidade e com maior frequência.^59^^,^^60^ Esse tipo de dieta tem se mostrado eficaz na diminuição dos sintomas ortostáticos em pacientes portadores de falência autonômica pura e atrofia multissistêmica. Existem evidências de que a dieta com baixo teor glicêmico pode causar um efeito benéfico em pacientes como sintomas de HO. A hipotensão pós-prandial também pode ser reduzida com o uso de cafeína ou acarbose.^61^

A anemia leva a uma diminuição da viscosidade sanguínea e da capacidade de carreamento de oxigênio, com aumento potencial dos sintomas de HO. Portanto, ela deve ser prevenida e tratada.^62^ O déficit de vitamina B_12_ pode estar associado a instabilidade postural e causar HO, sendo causa reversível de algumas polineuropatias.^63^ Dessa forma, as mudanças na dieta e a suplementação com vitaminas e ferro nos pacientes com deficiências destes minerais pode ser útil aos pacientes com HON.

### Manobras físicas para elevar a pressão arterial

Os pacientes com HO devem ser informados a respeito de medidas simples que podem ser utilizadas para aumentar a PA durante as atividades diárias. Entre as contra-manobras físicas, incluem-se o cruzamento de pernas, o agachamento e a tensão dos músculos das pernas, braços, abdômen, nádegas ou do corpo inteiro.^64^ Essas manobras geram aumentos da pré-carga cardíaca e, consequentemente, no débito cardíaco, na pressão arterial e na perfusão cerebral.^64^

A manobra mais básica é a ativação da bomba muscular das panturrilhas (musculatura “antigravitacional”). Se as válvulas venosas são competentes, a ativação muscular aumenta a pressão venosa cardíaca e de enchimento cardíaco. Pequenos aumentos na PA podem alterar a autorregulação e prevenir a pré-síncope e a síncope.^65^

Os pacientes devem ser alertados de que o ato de sentar ou deitar melhora os sintomas, ainda que estes possam recorrer após o retorno à postura ortostática. Existem evidências que sinalizam para o efeito benéfico da contração voluntária dos membros inferiores por 40 segundos após adoção da ortostase.^47^^,^^48^

O treinamento de contramanobras respiratórias que facilitem o retorno venoso do abdômen e dos membros inferiores para o coração também é útil, pois estas se utilizam de respiração profunda lenta e da criação de resistência inspiratória.

Muitos pacientes, particularmente os portadores de disautonomias mais graves, necessitam de ajuda de terceiros para realizar as contramanobras físicas. Eles devem ser aconselhados a levantar-se lentamente (ao longo de mais 15 segundos), uma vez que já foi comprovado que tal método atenua a queda da pressão arterial.

### Atividade física

A atividade física e o exercício devem ser incentivados a fim de evitar o descondicionamento, que sabidamente pode piorar a intolerância ortostática.^66^ Os mecanismos subjacentes à exacerbação estão relacionados à hipovolemia e ao remodelamento do ventrículo esquerdo, levando à deterioração do desempenho da câmara ventricular esquerda. Tais alterações cardíacas são revertidas pelo treinamento e aptidão físicas.^67^^–^^69^

Entretanto, a realização de exercício fisico, particularmente nos casos de HO por disautonomia, demonstrou que a adoção da postura ortostática, imediatamente após a prática de exercício em decúbito supino, pode exarcerbar a HO nesses pacientes. Tal observação não é reprodutível em indivíduos saudáveis.^67^^–^^69^

Por conta desse fator, principalmente nos pacientes idosos, portadores de HON, o exercício físico muitas vezes deve ser supervisionado por familiares ou profissionais especializados para evitar lesões ou quedas. Nesse subgrupo de pacientes, deve-se priorizar o treinamento físico moderado e, sobretudo, direcionado para os membros inferiores e exercícios físicos que não gerem um maior estresse gravitacional, como a bicicleta em decúbito supino ou exercícios em meio aquático.

Os pacientes devem evitar exercícios extenuantes, já que podem causar o aumento da temperatura corporal e a vasodilatação periférica, com consequente risco de hipotensão ortostática.^70^^,^^71^

Para minimizar a HO, o paciente deve ser hidratado antes e durante todo o período do exercício e ser alertado sobre o risco inicial de potencialização da HO logo após a interrupção do esforço físico.

### Evitar o aumento da temperatura corporal

A elevação da temperatura corporal causa vasodilatação periférica. Pacientes com HON devem evitar situações que causem aumento da temperatura corporal, como exercícios físicos de alta intensidade, exercícios em ambientes com alta temperatura e umidade, saunas ou banhos quentes.^72^ Além disso, indivíduos com falência autonômica, por terem um comprometimento da capacidade termorregulatória, apresentam maior risco de hipertermia.

### Cabeceira elevada

A elevação da cabeceira da cama é uma medida importante, realizada através do uso de uma cunha sob o colchão ou da colocação de blocos sob as pernas da cabeceira, para que a cabeça do paciente fique de 20 a 30 cm mais alta do que os pés, reduzindo a hipertensão supina. Ângulos de inclinação menores podem não ser tão bem-sucedidos, e travesseiros dobrados sob a cabeça podem não ser suficientes.

A hipertensão supina comumente leva ao aumento da noctúria e a depleção volêmica noturna. Este aumento da diurese noturna diminui com a elevação da cabeceira. Adicionalmente, ainda que modesto, o aumento do estresse gravitacional noturno mantém a ativação do sistema renina-angiotensina-aldosterona, o que permite uma pressão mais elevada durante a manhã.

A eficácia dessa intervenção foi questionada em um estudo randomizado recente que, entretanto, não diferenciou as causas de HO e não monitorou adequadamente a hidratação e o grau de elevação do leito, aspectos esses que podem ter contribuído para um resultado negativo. Consequentemente, recomenda-se que pelo menos nos pacientes com falência autonômica, seja orientada a elevação da cabeceira da cama. Tal postura específica não é isenta de efeitos adversos, podendo estar associada ao edema de tornozelo, ao deslizamento do corpo na cama e, como resultado, à dor nos pés.^73^^–^^75^

### Vestuário compressivo

O uso de meias elásticas que gerem um gradiente de pressão pode ser benéfico no tratamento da HO. Meias ou bandagens de compressão reduzem o acúmulo de sangue periférico nas extremidades inferiores, a hipotensão ortostática e os eventuais sintomas.

É preferível que a compressão se estenda até a cintura, pois a maioria da estase ocorre na circulação esplâncnica, que contém até 25% do volume sanguíneo em repouso. Deve-se colocar as meias pela manhã, com paciente deitado na cama e antes que ele se levante.

Esses procedimentos não invasivos costumam ser desafiadores, de baixa aceitabilidade e demandam a ajuda de terceiros, principalmente em pacientes idosos e com doenças neurológicas. Os benefícios a longo prazo dessas intervenções não foram estudados. Alguns autores sugerem que uma alternativa aceitável, devido à baixa adesão a longo prazo do vestuário compressivo, seria a utilização de roupas de ciclistas, que podem oferecer uma compressão abdominal satisfatória.

De qualquer forma, a associação entre as técnicas de compressão (particularmente do abdômen) e as contramanobras físicas se mostram muito efetivas nos pacientes portadores de disautonomia de etiologia neurogênica.

Um aumento da pressão abdominal de 20-40 mmHg através do uso de cintas abdominais e associada às contramanobras físicas de contração dos membros inferiores, resultam em um aumento significativo da resposta pressórica ao estresse gravitacional. Estudos que avaliaram o tratamento não farmacológico das disautonomias, identificaram evidências no uso de vestuário compressivo.^76^^–^^79^

## Tratamento farmacológico

A adição do tratamento farmacológico pode ser necessária em pacientes com HO grave, quando as abordagens não farmacológicas são insuficientes para prevenir os sintomas pré-sincopais ou sincopais (Figuras 3 e 4). É importante que se considere adequadamente o diagnóstico provável do paciente, como o HON, o SPOT ou a síndrome da fadiga crônica.

A presença de hipertensão prévia ou hipertensão supina, comum em pacientes com disautonomia, e a doença cardiovascular subjacente também devem ser consideradas. *O* tratamento da HO é desafiador por conta das poucas opções terapêuticas. Apenas a midodrina e a droxidopa (aprovadas nos EUA e no Japão) apresentam evidências de ensaios clínicos randomizados que apoiam o seu uso no tratamento de HO. Os dois fármacos normalmente não se encontram disponíveis no mercado brasileiro.

Não existem estudos comparativos que visem orientar a escolha inicial do medicamento na HON. A seleção de um medicamento ou outro, em muitas situações, estará relacionada à preferência, à experiência do clínico e a possibilidade de acesso ao medicamento por parte do paciente. Devemos sempre levar em conta a gravidade e a existência de comorbidades (especialmente a insuficiência cardíaca e/ou renal).

Esses agentes podem aumentar a PA e a volemia, o que pode agravar a hipertensão supina. Consequentemente, a melhora esperada da hipotensão ortostática (e a redução do risco de síncope e quedas) deve ser ponderada em relação aos riscos, a longo prazo, da hipertensão.

Outros desafios associados ao tratamento são a disponibilidade limitada de evidências clínicas e a falta de estudos comparativos de eficácia. A seguir, apresentamos uma visão geral dos principais medicamentos usados no tratamento da HO e as recomendações de uso.^79^

### Midodrina

O midodrina foi o primeiro fármaco aprovado pela agência reguladora americana (FDA) para o tratamento da HO. Trata-se de um pró-medicamento que pode ser rapidamente convertido em seu metabólito ativo, a desglimidodrina. É um agonista alfa-1-adrenérgico seletivo, com meia-vida curta (pico de ação após uma hora) e duração de ação estimado de 3-4 horas. Demonstrou-se que o midodrina aumenta significativamente a pressão sanguínea em ortostase e reduz os sintomas de intolerância ortostática.

Uma metanálise recente também concluiu que o midodrina melhora os resultados clínicos, com mínimos efeitos colaterais significativos. A dose geralmente começa em 2,5 mg, podendo chegar até a 10-15 mg por dose, em até 3 vezes ao dia. Devido a sua meia-vida curta, o esquema de dosagem típico é a cada 4 horas, a primeira ao acordar. A midodrina não deve ser administrado na hora de dormir e os pacientes devem evitar deitar-se por quatro horas após a última dose de midodrina, a fim de afastar o risco de agravamento da hipertensão supina.^79^^–^^81^

Dada a sua meia-vida curta, também pode ser usada de acordo com o necessário, antes de atividades específicas relacionadas a ocorrência de hipotensão ortostática sintomática. Os efeitos colaterais do midodrina são a hipertensão supina, a piloereção, o formigamento no couro cabeludo, a urgência ou retenção urinária e a cefaleia.

A midodrina está contraindicada em paciente que apresente doença cardíaca grave, bradicardia, história de angina, glaucoma de ângulo fechado, doença arterial oclusiva grave, tireotoxicose, feocromocitoma, insuficiência renal grave, doença de Raynaud e retinopatia diabética proliferativa. Deve-se, também, estar atento aos pacientes com insuficiência cardíaca e insuficiência renal crônica.

### Fludrocortisona

Em pacientes sem hipertensão ou insuficiência cardíaca, a fludrocortisona é incluída no tratamento baseada na opinião de especialistas, sendo mais amplamente usada em países que não dispõem dos outros fármacos preconizados. A fludrocortisona é um mineralocorticóide sintético que aumenta o volume intravascular e a reabsorção renal de sódio. Os efeitos da fludrocortisona a longo prazo na PA, no entanto, são atribuídos a maior sensibilidade dos vasos sanguíneos a noradrenalina e a angiotensina II. A dose inicial típica é de 0,05 mg por dia e pode ser aumentada até 0,3 mg (em dose única ou fracionada).

O início de ação ocorre no período de três a sete dias. Seus efeitos colaterais podem incluir hipocalemia, dores de cabeça, edema periférico, insuficiência cardíaca e hipertensão supina. Em doses mais altas, os pacientes podem apresentar risco aumentado de supressão do eixo hipotálamo-hipófise-adrenal. Cerca de 30% dos pacientes deixam de usar o medicamento por conta dos efeitos colaterais.

De modo geral, em pacientes com hipertensão supina preexistente, a fludrocortisona geralmente não é escolhida como medicamento de primeira linha, sendo a midodrina o mais apropriado. A evidência clínica formal que apoia o uso do fármaco para o tratamento da HO neurogência é escassa.^82^^–^^84^

### Droxidopa

Mais recentemente, o FDA aprovou a droxidopa para o tratamento da hipotensão ortostática neurogênica nos Estados Unidos, especialmente na doença de Parkinson, na atrofia multisistêmica e na falência autonômica pura. A droxidopa é um pró-fármaco sintético que se converte em noradrenalina no cérebro e nos tecidos periféricos. Os níveis circulantes de norepinefrina aumentam no período máximo de seis horas após a droxidopa. A droga tem pico plasmático entre 1-4 hs, com média de 2hs em indivíduos saudáveis.

A droxidopa é bem tolerada e melhora a tolerância ortostática em ensaios controlados em HON (100-600 mg VO, 3 vezes ao dia). Semelhante ao midodrina, a droxidopa não deve ser administrada dentro de cinco horas antes de dormir. Recomenda-se cautela no uso do medicamento em pacientes com insuficiência cardíaca congestiva e insuficiência renal crônica. Seus efeitos colaterais incluem dor de cabeça, tontura, náusea e fadiga.^85^ Interações importantes existem com os fármacos usados para tratamento da doença de parkinson.

### Outros medicamentos

Outros medicamentos são a pseudoefedrina, a atomoxetina (inibidor da recaptação de noradrenalina), a ioimbina (antagonista do receptor alfa-2-adrenérgico), a octreotida (análogo da somatostatina), a ergotamina, a eritropoetina e a piridostigmina (inibidor da colinesterase).^86^^–^^89^

A *atomoxetina* é um inibidor de transportador de noradrenalina, aprovado para o tratamento do transtorno do déficit de atenção e hiperatividade (TDAH). No entanto, em pacientes com insuficiência autonômica que apresentem função noradrenérgica periférica intacta, ela pode causar uma vasoconstrição periférica potente e, consequentemente, o aumento da pressão arterial. Esse medicamento é pouco eficaz na falência autonômica pura (FAP), por conta do comprometimento periférico do sistema noradrenérgico.

A *piridostigmina*, um inibidor da acetilcolinesterase que aumenta a disponibilidade da acetilcolina nas terminações nervosas, agiria na prevenção da HO. Entretanto, ela aumenta a atividade nervosa simpática em resposta ao estresse ortostático, causando uma mudança na sensibilidade do baroreceptores. Provavelmente, ela é mais útil em pacientes de menor gravidade, com função simpática residual e possui a vantagem de não piorar a hipertensão supina. Estudos mostram que a menor eficácia da piridostigmina se comparada a fludrocortisona na HO da doença de Parkinson, ainda que cause menor hipertensão supina e apesar do aumento da hipertensão supina periférica não ser acompanhado de similar aumento na pressão arterial central com a fludrocortisona.^90^^,^^91^

A *acarbose*, agente que impede a absorção de glicose no intestino delgado, diminui a liberação de hormônios gastrointestinais e retarda o esvaziamento gástrico quando administrada 20 minutos antes da refeição. Esse padrão tem se mostrado eficiente nos casos de hipotensão pós-prandial. Ela é contraindicada em pacientes com cetoacidose diabética, cirrose, doença inflamatória intestinal, colite ulcerativa, obstrução intestinal ou qualquer doença intestinal crônica que possa interromper a digestão ou absorção.

A *cafeína* (200-250 mg ou uma xícara de café de 200 mL por dia), quando administrada nos pacientes que não são usuários crônicos, pode ajudar a inibir a vasodilatação periférica e, portanto, é capaz de aumentar a pressão arterial em ortostase.

Um estudo recente mostrou que a *di-hidroergotamina*, em combinação com a cafeína, pode ser utilizada como tratamento alternativo em pacientes com falência autonômica e sem doença arterial coronariana vascular subjacente.^87^

## Tratamento farmacológico combinado

Existem poucos dados para determinar a eficácia e a segurança de diferentes combinações de terapia em comparação à monoterapia para a HO. Recomenda-se buscar a dose máxima tolerável de um único agente e, em seguida, caso o benefício sintomático não seja obtido, considerar a troca para uma terapia diferente ou adicionar um segundo agente e titular, a partir da menor dose efetiva.

A associação mais comum nos casos refratários se dá entre a midodrina e a fludrocortisona. A utilização de água, sal e de medidas preventivas já discutidas também podem ser efetiva quando combinada aos fármacos. Uma combinação entre os demais fármacos é possível, sempre com atenção a flexibilidade de dose (particularmente dos fármacos de meia-vida curta, como o midodrina) e o controle rigoroso da adesão ao tratamento não farmacológico.^92^^,^^93^

## Peculiaridades no manejo da hipertensão supina e hipotensão pós-prandial

### Hipertensão supina e noturna

Nos pacientes portadores de HO, especialmente HON, é frequente observarmos a associação a hipertensão supina e noturna, sendo que a gravidade da hipertensão noturna se correlaciona com a magnitude da HO. A hipertensão supina é distinta da hipertensão essencial, uma vez que a maioria dos pacientes é normotenso enquanto está sentado e pode ser severamente hipotenso em pé. Aproximadamente 50% dos pacientes com FAP apresentam hipertensão supina. A avaliação da hipertensão supina e noturna deve ser realizada de rotina nos pacientes com HON, pois a sua presença é um limitador das opções terapêuticas, devido a possibilidade de efeitos adversos. A MAPA também pode ser utilizada na avaliação diagnóstica e para o seguimento clínico.

Na maioria dos pacientes, existem fortes razões para priorizar o tratamento da HON sobre a hipertensão supina. A HO sintomática é a causa de uma variedade de sintomas relacionados à postura, incluindo tonturas, pré-síncope ou síncope, fadiga, dor cervival em nuca, fraqueza e deficiência visual durante ortostase. Todos os sintomas que podem contribuir para um aumento na ocorrência de quedas devem ser bem avaliados, pois estas representam uma das mais comuns causas de internação hospitalar, com elevada morbimortalidade.

Para a prevenção e o tratamento da hipertensão supina, deve-se:

1. Dormir com a cabeceira da cama elevada;
2. Consumir refeição rica em carboidratos na hora de dormir;
3. Evitar a ingesta de líquidos antes de dormir;
4. Evitar o decúbito supino durante o dia, especialmente nos pacientes que estejam usando vestuário de compressão ou fármacos vasopressores.

Não existem medicamentos aprovados para o tratamento da hipertensão supina, mas existem vários agentes potencialmente úteis. Em pacientes que ainda apresentam algum tônus simpático, um agonista alfa-2 central (clonidina) reduz o fluxo simpático quando administrado ao final da tarde, sem exacerbar a hipotensão ortostática durante o dia. É importante evitar-se o uso de diuréticos e anti-hipertensivos de ação prolongada, mesmo que eles possibilitem o controle da hipertensão supina.^89^^,^^92^^,^^93^

**Tratamento para hipertensão supina na disautonomia**

O ponto de corte da PA para iniciar a terapia antihipertensiva não foi definido e as decisões de tratamento devem ser tomadas individualmente. No entanto, anti-hipertensivos podem ser indicados com cautela se a PA noturna atingir valores predominantemente ≥ 160/100 mmHg (Tabela 2), com o uso de fármacos de ação curta.^89^^,^^92^^.^^93^

A hipertensão supina é distinta da hipertensão essencial, uma vez que a maioria dos pacientes é normotensa enquanto está sentada e pode ser gravemente hipotensa em pé.

**Tabela 2:** Tratamento da hipertensão arterial supina

Fármacos*	Mecanismo de ação	Dose habitual
Captopril	Inibidor da enzima de conversão de angiotensina	25 mg à noite
Clonidina^a^	Agonista alfa-2 central	0,1-0,2 mg após refeição noturna
Hidralazina	Relaxamento da musculatura lisa periférica	10-25 mg à noite
Losartana	Antagonista dos receptores de angiotensina II	50 mg à noite
Nitroglicerina (patch)	Vasodilatador	0,1 mg/h (patch – remover pela manhã)

* Os medicamentos anti-hipertensivos de meia-vida curta devem ser os de uso preferencial para o tratamento da hipertensão supina. A administração deve ser feita apenas no período noturno. Lembrar que muitos desses medicamentos possuem dosagem habitual de 2-3x/dia. Quando tomados inadvertidamente dessa forma ou durante a vigília, podem piorar os sintomas de HON

a O uso da clonidina aumenta o risco de hipotensão matinal.

### Hipotensão pós-prandial

A hipotensão pós-prandial (HOP) é comumente observada em portadores de HO, mas pode ocorrer isoladamente, em particular nos pacientes idosos institucionalizados. Os mecanismos que levam ao declínio da pressão arterial não são claros. A ocorrência de hipotensão pós-prandial e a sua extensão são favorecidas pela ingestão de glicose.

As estratégias de tratamento incluem: refeições pequenas, frequentes e com baixo conteúdo de carboidratos; beber água antes e durante a refeição (recomenda-se tomar 400-500 mL de água gelada, 30 minutos antes das refeições); minimizar ou, preferencialmente, evitar a ingestão de álcool; eliminar as causas iatrogênicas (administração de anti-hipertensivos entre as refeições, não durante as refeições) e o uso de cafeína (200-250 mg ou uma xícara de café de 200 mL) e a acarbose (100 mg).
