# Supplementary material for: Disautonomia: Uma Condição Esquecida – Parte II
Source: Arq Bras Cardiol. 2021 May 6;116(5):981–98. [Article in Portuguese] doi: 10.36660/abc.20200422 (PMC8121459; doi:10.36660/abc.20200422)
Supplement: Translation

# Translation: Dysautonomia: A Forgotten Condition — Part II

**Authors:** Eduardo Arrais Rocha, Niraj Mehta, Maria Zildany Pinheiro Távora-Mehta, Camila Ferreira Roncari, Alan Alves de Lima Cidrão, Jorge Elias

**Affiliations:** 1 FortalezaCE Brazil Hospital Universitário Walter Cantídio da Universidade Federal do Ceará (UFC) - Programa de Pós-graduação em Ciências Cardiovasculares da Faculdade de Medicina da UFC, Fortaleza, CE - Brazil; 2 CuritibaPR Brazil Universidade Federal do Paraná, Curitiba, PR - Brazil; 3 CuritibaPR Brazil Clínica de Eletrofisiologia do Paraná, Curitiba, PR - Brazil; 4 FortalezaCE Brazil Departamento de Fisiologia e Farmacologia - Faculdade de Medicina da Universidade Federal do Ceará (UFC), Fortaleza, CE - Brazil; 5 FortalezaCE Brazil Programa de Pós-graduação em Ciências Cardiovasculares da Faculdade de Medicina da UFC, Fortaleza, CE - Brazil; 6 VitóriaES Brazil Serviço de Eletrofisiologia do Vitória Apart Hospital, Vitória, ES - Brazil

**Keywords:** Primary Dysautonomias, Hypotension, Orthostatic, Hypothension, Postural, Fatigue, Diabetes, Autonomic Nervous System, Syncope, Cardiovascular Autonomic Neuropathy

## General and Cardiovascular Clinical Manifestations

The pathologies that affect the autonomic nervous system (ANS) can manifest in different ways, depending on the etiology, degree of impairment, disease duration, presence of comorbidities, age or use of associated drugs. Many symptoms can be completely debilitating, such as severe pain in peripheral neuropathy and falls or syncope in autonomic neuropathies. Progression to severe orthostatic intolerance may occur in more advanced cases of dysautonomies, with severe early orthostatic hypotension and supine hypertension, making treatment difficult (Table 1).^1^^–^^7^

**Table 1:** Clinical and Cardiovascular Signs and Symptoms of Dysautonomia and/or Cardiovascular Autonomic Neuropathy (CAN)

Clinical symptoms	Cardiovascular signs and symptoms
Impotence in men and reduced libido	Fatigue and exercise intolerance
Abnormal menorrhea	Pre-syncope/syncope
Urinary urgency and incontinence	Visual darkening and intolerance to prolonged orthostasis
Diarrhea/Constipation/Indigestion	Unexplained falls
Exacerbated responses to hypoglycemic agents	Exacerbated responses to antihypertensive drugs
Difficult control of diabetes (due to gastroparesis)	Supine hypertension
Hypohidrosis or anhidrosis	Non-dipper pattern on Ambulatory Blood Pressure Monitoring (ABPM)
Abnormal vision, pupil atrophy	Tiredness, shortness of breath (due to chronotropic incompetence)
Pain, numbness or burning feeling in the extremities	Bradycardia
Forgetfulness, decreased cognitive function	Paleness, cold extremities
Tremors, unbalance	Orthostatic hypotension
Sleep abnormalities/apnea	Postprandial syncope or pre-syncope (up to 2 hours after a copious or high-carbohydrate meal)
Severe pain in the posterior cervical region (trapezius ischemia)	Palpitations and tachycardia on rising

Source: Author.

## Investigation methods

The Autonomic Nervous System (ANS) is reasonably complex, which makes it hard to investigate it and interpret it at first. However, some tests are simple, easy to perform and provide valuable information about its shortcomings. They can be performed using modern computerized equipment or through simple digital electrocardiograms that can record the tests, the RR intervals, allowing for adequate measurements of the relationships between their variations.

The objectives of this evaluation are:

- To confirm diagnosis;
- To stage the severity of dysfunction;
- To identify subclinical abnormalities;
- To monitor evolution of the disease.

For the effective performance of autonomic tests, the patient must be rested and calm. The autonomic evaluation laboratory should be a quiet properly heated and lightly darkened place.^8^^,^^9^

### Cardiovascular Autonomic Reflex Tests (Cardiovagal Function Tests)

These tests were described by Ewing, in the 70s, and today, they are the gold standard tests for the diagnosis of cardiovascular autonomic neuropathy (CAN).^7^^,^^10^^-^^13^ They have good sensitivity and specificity and must be performed in the presence of symptoms suggestive of dysautonomia, and early in patients with pathologies that include diabetes, which may progress to CAN, even in their glucose intolerance phase (Figure 1).^8^^–^^10^

**Figure 1:** Cardiovascular Autonomic Reflex Tests. Clinical case of a patient with advanced disautonomia with abnormal breathing tests (1.08) and abnormal orthostatic coefficient 30/15 (1.03). Values need to be adjusted for age and sex.^11^^–^^13^^.^

The tests are divided into methods that assess sympathetic and parasympathetic function, which are usually abnormal earlier, especially in diabetes.

Abnormal results in one method of the 3 cardiovascular tests implies early or uncertain autonomic neuropathy. The test must be repeated after 1 year for confirmation and evaluation of evolution. The presence of 2 positive tests is confirmatory for CAN. The association of orthostatic hypotension with 2 positive tests implies advanced dysautonomia and worse prognosis.

These tests require proper assessment and preparation, with the suspension of several drugs that can alter the analysis of heart rate and ANS. Proper evaluation of this test is not possible in patients with frequent arrhythmias (more than 6 ectopic beats per minute), atrial fibrillation, cardiac pacemaker, and accentuated tremors and non-collaborative patients.

**Cardiovascular Autonomic Reflex Tests (Cardiovagal Function Tests)**

These were described by Ewing in the 70s. They are the gold standard tests in the diagnosis of Cardiovascular Autonomic Neuropathy (CAN). They have good sensitivity and specificity and should be performed in the presence of symptoms suggestive of dysautonomia, and early in patients with pathologies such as diabetes, which may progress to CAN, even in their glucose intolerance stage.^7^^,^^11^^-^^13^

They can be performed using modern computerized equipment or through simple digital electrocardiograms, which can record the tests, the RR intervals, allowing for adequate measurements of the relationships between the variations of the longest and shortest RR intervals.^14^^–^^16^

### Breathing Test (E/I Respiratory Quotient)

This method analyzes the ratio (quotient) between the highest RR cycle on exhalation divided by the largest RR cycle on inspiration — 3 cycles of 1 minute each are performed, with 1-minute intervals between tests, allowing to evaluate the parasympathetic system.

Inhalation and exhalation cycles are slow and deep, with a total respiratory cycle lasting 10 seconds. It accentuates the sinus respiratory arrhythmia seen in normal individuals. The normal response is an acceleration of the heart rate during inspiration and deceleration on exhalation. In summary, heart rate is recorded for 1 minute (6 slow, deep breathing cycles lasting 10 seconds each). The difference between the maximum and minimum heart rate, or the ratio of these two (E:I ratio), is recorded and measured in milliseconds.

Usually, respiratory amplitudes are averaged over the 6 cycles. This is a test that assesses parasympathetic response to respiratory stimulus. Patients with dysautonomia may experience reduced or no heart rate oscillation on deep breathing. Loss of respiratory sinus arrhythmia may be one of the first signs of diabetic autonomic neuropathy.

Normal physiological values of the amplitude difference are considered above 15 bpm. Between 11–14 bpm, borderline and below 10 beats would be pathological. The E:I ratio (maximum heart rate measured in milliseconds during exhalation divided by the maximum heart rate during inspiration) in normal individuals must be greater than 1.2.^11^^–^^15^ These values must be adjusted for age and sex.

### Valsalva Test — (Valsalva Quotient)

In this test, the relationship between the largest RR cycle in the relaxation phase divided by the largest RR cycle in the Valsalva maneuver phase is measured, allowing to assess particularly the parasympathetic system and also the sympathetic system, when associated with continuous blood pressure measurements.

The Valsalva maneuver is particularly interesting because it tests the integrity of both the cardiovagal parasympathetic response, by analyzing heart rate, and the sympathetic response, by analyzing blood pressure. The technique basically consists of making the monitored patient blow for 15 seconds through a small tube, with a discreet air outlet to avoid closing the glottis. The expiratory air pressure generated should be around 40 mmHg.

There are 4 distinct phases: blood pressure deflections in phases I and III represent mechanical disturbances generated by changes in intrathoracic pressure, at the beginning and at the end of the Valsalva maneuver. On the other hand, phase II and phase IV are the clinically relevant phases.

In healthy individuals, during expiratory effort, in phase I, blood pressure drops due to decreased venous return. This pressure drop is perceived by intact baroreceptors, which trigger a response with increased sympathetic tone, leading to vasoconstriction and increased heart rate.

This action recovers pressure in late phase II. On release of intrathoracic pressure at the end of the maneuver, there is increased venous return and, with the maintenance of peripheral vasoconstriction, there is blood pressure overshoot (perfect combination of increased venous return and vasoconstriction).

Patients with autonomic dysfunction are unable to react with increased sympathetic tone to the initial pressure drop caused by the maneuver. Therefore, there is no pressure increase in late phase II and, typically, there is no blood pressure overshoot in phase IV. Instead, pressure response in patients with dysautonomia reveals a gradual return of blood pressure to baseline levels after induction of hypotension caused by the forced expiration.^17^^–^^19^

With regard to heart rate, the Valsalva quotient is derived from the maximum heart rate measured in milliseconds and generated by the Valsalva maneuver, divided by the lowest heart rate in the first 30 seconds of the peak heart rate. Heart rate responses are mediated by baroreceptors.

Increase in heart rate is due to the blood pressure drop. Besides, baroreceptor response to the blood pressure overshoot in phase IV is responsible for the transient bradycardia at the end of the maneuver.^17^^–^^19^

In patients with dysautonomia, there is a loss of both blood pressure overshoot and reflex bradycardia. Therefore, heart rate does not respond either, due to the absence of increased sympathetic response, showing a straight curve (absence of heart rate oscillation).

The normal Valsalva quotient (maximum RR value in ms divided by the lowest RR value) during the maneuver must be greater than 1.21. Borderline values would be between 1.11 and 1.20, while pathological values would be considered if less than or equal to 1.10.^13^^–^^15^ These values must also be adjusted for age and sex.

### 30/15 Quotient Test with Orthostasis

RR interval is evaluated after orthostasis around the 15^th^ beat (usually higher frequency — lower RR interval) and around the 30^th^ beat (lower frequency — higher RR interval), indicating a predominant evaluation of the parasympathetic system.

The simplest and most commonly used method for testing cardiovascular feedback is by measuring cardiovascular parameters (heart rate, blood pressure, noradrenaline dosage) during postural change from horizontal posture to orthostatic posture.^16^^–^^19^

Due to changes in hydrostatic pressure, when standing, 500–800 ml of volume is redistributed to the lower limbs. However, when actively standing the lower limb veins are compressed (the so-called skeletal-muscle pump), immediately increasing venous return.

Compensatory mechanisms act quickly, causing blood pressure to change very little in healthy individuals. However, in 10 to 15% of individuals, orthostatic circulatory disorders are observed due to insufficient compensatory mechanisms.

Evaluation of response to orthostasis can be performed by active inclination or by response to the tilt test. In the first, with regard to heart rate, there is a rapid and maximum increase around the fifteenth heart beat in normal people. After that, there is relative maximum bradycardia around the thirtieth beat. Pharmacological studies indicate that this response is mediated by the vagus nerve.

Patients with diabetes-associated cardiovascular autonomic neuropathy show only a slight progressive increase in heart rate.^19^ The 30:15 ratio (or Ewing's ratio) is used as a measure of parasympathetic integrity. The longest RR interval at the 30^th^ beat and the shortest RR interval at the 15^th^ beat is called the Ewing ratio or 30:15 ratio, where a normal value would be above 1.04.

Current software packages no longer calculate pure 30:15 ratio. Instead, they use the measurement of the longest RR interval between the 20^th^ and 40^th^ heartbeat and the shortest RR interval, between the 5^th^ and 25^th^ heartbeat.^14^^–^^19^

## Protocol of the 7 Tests for Dysautonomia

Association of 3 cardiovascular function tests with 3 tests of analysis of RR variability in the frequency domain and orthostatic hypotension investigation represents the protocol of the 7 tests (figures 1 and 2) for investigation of CAN, with high sensitivity and specificity.

**Figure 2:** **Analysis of RR Variability in the frequency domain** during a 5-minute cycle. Any drugs that interfere with heart rate analysis must be discontinued. The presence of frequent arrhythmia, atrial fibrillation or pacemaker makes it impossible to analyze the test. **Very low frequency component — 0.01–0.04 Hz (FMB – VLF) —** Assessment of vasomotor tone fluctuations related to thermoregulation and sweating — (predominant sympathetic action) **Low Frequency Component — 0.04–0.15 Hz (FB-LF) —** Baroreceptor evaluation (predominant sympathetic component with vagal modulation) **High Frequency Component — 0.15–0.5 Hz (FA — HF) —** Related to sinus control (parasympathetic action) **It can be carried out in the protocol with the tests in** **figure 1** **(characterizing the protocol of the 7 Cardiovascular Autonomic Reflex Evaluation Tests)**.

This test may have a better diagnostic capacity according to some authors.^12^ It is considered positive if 3 methods out of the 7 tests are abnormal, or uncertain or early if 2 abnormal methods. Similar to the isolated cardiovagal tests, the presence of associated orthostatic hypotension implies greater CAN severity.

These tests can be performed together using specific software such as the VNS-Rhythm poly-spectrum analysis^®^ or Neuro-Diag. Ansar^®^, which are inexpensive compared to equipment used with hemodynamic measures.

## Tilt test

The tilt test is a very useful diagnostic tool for patients with dysautonomia. On orthostatic position, 500–800ml of central volume is transfered to the periphery (pelvis, abdomen and lower limbs). This volume movement leads to a drop in systolic volume and, consequently, cardiac output. This drop, in turn, is felt by the baroreceptors of the aortic arch and carotid sinus which, after interaction with vasomotor centers, trigger a response with a reduction in parasympathetic activity and an increase in sympathetic activity. This translates into peripheral vasoconstriction and increased heart rate.

This diagnostic test can be particularly useful to detect and confirm the autonomic failure seen in orthostatic hypotension, orthostatic postural tachycardia, late orthostatic hypotension and, obviously, reflex changes in vaso-vagal reaction.^19^^–^^20^

The tilt test is performed in a quiet room with minimal distractions. The patient is initially instructed to fast for 3 hours and lie down in the supine position for at least 10–15 minutes. Although there are several protocols, the current recommendation and the most commonly used protocol is inclination at 70 degrees for about 30–40 minutes.^21^

Provocative test with isoproterenol or 1.25 mg sublingual isosorbide can be useful for investigating syncope of vaso-vagal origin as it increases test sensitivity.^22^ However, drug sensitization does not apply when the goal is to evaluate dysautonomia, because it is necessary to evaluate spontaneous cardiovascular physiological response to prolonged orthostatic stress.

Although intermittent blood pressure measurements (every 2–3 minutes) can be performed, continuous monitoring of blood pressure and electrocardiographic recording would be preferable, however at a much higher cost.

## Tilt test associated with hemodynamic measurements

The use of additional modules to measure continuous blood pressure (Finapress^®^ modelflow and Task Force Monitor^®^ impedance cardiography) allows to determine stroke volume indirectly. From this parameter, other hemodynamic parameters can be estimated with reasonable precision, such as peripheral vascular resistance and cardiac output.

Finapress^®^ modelflow uses the analysis of arterial pulse contour, which is a technique for determining stroke volume. In modelflow, the arterial pulse flow wave is calculated from the arterial pulse pressure contour. Integration of this flow wave with each beat generates the stroke volume.^22^^,^^23^

Impedance cardiography (Task Force Monitor^®^^®^), on the other hand, measures abnormalities in chest impedance generated by fluctuating blood volumes during the cardiac cycle, allowing to calculate stroke volume, cardiac output and other parameters.^23^ Although these noninvasive cardiac output determination techniques are not completely accurate, they have been validated compared to invasive techniques and are quite reliable to monitor the relative changes in cardiac output.

Therefore, the tilt test with hemodynamic parameters, in addition to measuring continuous blood pressure, allows to evaluate stroke volume, cardiac output and peripheral vascular resistance. Analysis of hemodynamic parameters during the tilt test is very important, as it allows to record the drop in peripheral vascular resistance and reveal the presence of dysautonomia, often without a significant drop in blood pressure due to borderline compensatory mechanisms (mild to moderate dysautonomia).

Besides, it allows to determine whether there is a significant reduction in stroke volume, which may be a non-neurogenic component of orthostatic hypotension (due to chronic dehydration, for example).

Thus, the tilt test with hemodynamic parameters allows to identify subclinical abnormalities in ANS integrity even without any evident pressure drop, hence increasing test sensitivity. The limitation of the method is its high cost. It is restricted to few centers, especially for dysautonomia research.

## 24 hours Ambulatory Blood Pressure Monitoring (ABPM)

Daytime and nocturnal autonomic balance does not only affect heart rate, but also blood pressure. Normally, blood pressure fluctuates, with higher levels during wakefulness, declining at night (nocturnal decrease). Proportional blood pressure drops at night based on the daytime period determine the expected nocturnal decrease responses: attenuated response (0–10% nocturnal decrease), normal response (10–20% nocturnal decrease), marked response (above 20% decrease) and reverse response (increase in blood pressure instead of expected nocturnal decrease).

Attenuated or reverse response shows exacerbated sympathetic activity, which may be present in dysautonomia, and has been associated with increased mortality. In addition, the presence of nocturnal hypertension may increase the risk of daytime hypotension (as a result, among other things, of increased nocturnal excretion of natriuretic hormone).

ABPM can indicate cardiovascular autonomic dysfunction abnormalities and is able to select patients for a deeper assessment of dysautonomia. More specifically, ABPM can be especially useful in detecting nocturnal hypertension (an important predictor of cardiovascular events) and forms of early or postprandial orthostatic hypotension, usually not detected with the usual blood pressure measurements.

**Supine Hypertension – Warning Sign**

The presence of supine hypertension or non-dipper pattern on 24-h ABPM, especially in patients with pathologies that are known to involve dysautonomia must consider suspicion and investigation with clinical and laboratory screening for dysautonomia.

## Holter Monitoring and RR Variability Analyses

The sinus node is subject to both sympathetic and parasympathetic (vagal) action, depending on the situation evaluated. Standing position, mental stress and exercise are associated with increased sympathetic tone. On the other hand, vagal tone is increased in resting conditions. In normal individuals, both sympathetic and parasympathetic tone fluctuate throughout the day, generating a variation in RR intervals or simply RR variability. In normal individuals, RR variability declines 3–5 beats every decade.

24-h Holter can be used to analyze average heart rate, chronotropic incompetence and cardiac arrhythmias, and when coupled with specific software, it allows analyzing RR variability.

High mean heart rates may suggest autonomic dysfunction, such as in diabetic patients, or indicate inappropriate sinus tachycardia (IST), or even allow the identification of chronotropic incompetence. The detection of arrhythmias may suggest other etiologies as a justification for the symptoms, and helps in the selection for the performance of cardiovascular tests.

There are several methods for analyzing RR variability data, including time and frequency domain analysis. In the time domain analysis, each QRS is detected to determine the “normal to normal” interval. This interval also provides additional information, including standard deviation. More complex statistical analyses require extended periods of time. Spectral analysis can provide evaluation in the frequency domain, giving information on how variance is arranged as a function of frequency.

Heart rate abnormalities occur continuously during daily activities, reflecting autonomic balance, reflex cardiovascular mechanisms and external stimuli. In normal people, increased RR variability of heart rate is considered a measure of autonomic integrity, while reduced heart rate variation is an early sign of autonomic imbalance.

Analysis of RR variability can be done in the time domain and in the frequency domain for short periods of a few minutes or longer periods (24-hour Holter). Time domain analysis includes evaluation of many parameters, such as: mean normal intervals; mean heart rate, difference in maximum heart rate, standard deviation of the average normal-to-normal intervals (SDANN); root mean square of successive differences between normal heartbeats (rMSSD).

Prolonged monitoring (24-h Holter) also allows to calculate the number of instances per hour in which a difference greater than 50 ms was measured between two consecutive RR intervals (pNN50). Essentially, all of these indexes explore parasympathetic activity.

The spectral analysis of RR variability (frequency domain), in turn, reveals 3 main frequency components:

Very low frequency component (<0.04Hz) related to fluctuations in vasomotor tone linked to thermoregulation and sweating (sympathetic control);

Low frequency component (0.04–0.15 Hz) connected to the baroreceptor reflex (sympathetic control with vagal modulation);

High frequency component (0.15–0.4 Hz) influenced by breathing (respiratory sinus arrhythmia), being a component of parasympathetic activity.

In diabetic patients with predominantly vagal (earlier) dysfunction, the range of high frequencies is reduced or absent. On the other hand, in later sympathetic dysfunctions, the amplitudes of low and very low frequencies are reduced.

Time domain parameters, total spectral power of RR variability and the high frequency spectral component are parasympathetic parameters. Although the low frequency component is controlled by sympathetic activity, extreme sympathetic activation (as in exercise, in heart failure) attenuates RR variability, making it difficult to record it, and may, therefore, not correlate with the real sympathetic activity.

Thus, it is now accepted that the absolute “spectral power” of low frequencies does not reflect sympathetic activity. However, when measured in relative terms (as a percentage of the overall RR variability), the relative ratio of low to high frequencies provides a relative and approximate indication of sympathetic modulation of the heart.

Therefore, the ratio of low to high frequency component best represents sympathetic status. As RR variability is influenced by age, sex and respiratory rate, adjustment for these variables is recommended. Results of spectral analysis correlate well with the tests of autonomic function in clinical situations.

Spectral analysis is more sensitive in the early stages of CAN. In diabetic patients in the early stages of CAN, a progressive deterioration of the parameters of spectral analysis related to the parasympathetic system is documented. The expected nocturnal increase in the high frequency band of RR variability, representing vagal modulation of the heart, seems to be the earliest abnormality detected. During advanced stages of CAN, all components are eliminated.

## Electromyography and Small Fiber Neuropathies

Postganglionic autonomic fibers are type C, non-myelinated. With the Aδ fibers, little myelinated, they make up the group of fine fibers. They differ from thick myelinated fibers by their thickness and conduction speed: they conduct nerve impulses at a speed of 0.5 to 1 m/s, while in the latter, speeds of up to 120 m/s are observed.^24^^,^^25^

Aδ fibers are of the somatic sensory type and participate in skin innervation, mediating the perception of pain and thermal stimuli, whereas type C autonomic fibers innervate the cardiac musculature, smooth muscles (present in the blood vessel wall, gastrointestinal and genitourinary tracts) and salivary, lacrimal and sweat glands.^24^

Neuropathic involvement of small fibers can occur without the involvement of thick fibers, characterizing small fiber neuropathy, or in the context of a polyneuropathy, where there is a clear involvement of those. In the context of small fiber neuropathies, involvement of Aδ fibers is more commonly observed.

Typical symptoms include paresthesia, pain, a burning sensation or cold, with clear worsening at night. Dysautonomic signs and symptoms occur in approximately 50% of these patients. More rarely, small fiber neuropathy may appear predominantly with autonomic symptoms.^26^^,^^27^

Skin biopsy and quantitative sensory testing (QST) are useful mainly for the assessment of Aδ fibers. Other tests are for the evaluation of autonomic fibers and, when performed, they increase diagnostic sensitivity.^26^^–^^31^

Conventional electroneuromyography, through conduction and electromyography studies, is a key test for the initial evaluation of these cases, not to confirm diagnosis, but mainly to rule out polyneuropathy (involvement of thick fibers) and conditions that may appear like small fiber neuropathy, such as bilateral S1 radiculopathy which characteristically involves paresthesia of the feet.

Conduction studies by this method evaluate only the fastest nerve fibers and is not able to identify impairment of small fibers. Therefore, in pure cases of small fiber neuropathy, conduction studies, including evaluation of sural nerves, which are classically altered in cases of polyneuropathies, will be normal.^26^^,^^27^^,^^31^^,^^32^

Skin biopsy is still considered the gold standard for the diagnosis of small fiber neuropathy. It is a low-invasive procedure, performed on an outpatient basis, with local anesthesia. In general, a 3-mm tissue fragment is removed from the distal region of one of the lower limbs, 7 to 10 cm proximal to the lateral malleolus. Other fragments can be removed 7 to 10 cm proximal to the knee and 7 to 10 cm distal to the greater trochanter, of the same limb, to define the pattern — length-dependent (distal to proximal pattern) or not length-dependent, or biopsy of specific sites if focal symptoms are detected.

As mentioned before, evaluation of the density of intra-epidermal fibers predominantly focuses on the Aδ fibers.^28^ The expected normal values vary according to age, gender and biopsy site. Recent studies have sought to standardize these values.^33^^,^^34^ When the values are unknown for a given site, comparative analysis with the contralateral side may be a valid alternative.

Limitations of this method are: difficulty accessing specialized laboratories, especially in developing countries such as Brazil; the possibility that the test may still be normal at the beginning of the condition; no standardization of expected values for many anatomical sites and no standardization for the evaluation of autonomic fibers.

## Quantitative Sensory Testing and Sudomotor Test

The Quantitative Sensory Testing (QST Testing) assesses the pain sensation threshold mainly through controlled thermal stimulus, usually by heat. It therefore assesses the integrity of Aδ fibers. It is a test that depends on the patient's collaboration, since they need to be able to share their perceptions. Thus, it can be distorted by the difficulty of understanding instructions, patient's difficulty to concentrate or even by volitional action.

Other limitations to the method are: low availability and the fact that it does not distinguish peripheral from central injuries. Involvement of the spino-thalamic pathways and brain areas related to this sensory modality will also lead to an abnormal pattern in the test. For these reasons, it is not recommended that it be used as a single test to diagnose small fiber neuropathy.^26^^,^^30^

Tests of the autonomic fibers are mainly directed to the evaluation of sudomotor function. Sweat production by the sweat glands is mediated by cholinergic sympathetic innervation. It is observed, in general, in the presence of impairment of such fibers in a length-dependent pattern (distal to proximal), anhidrosis in the distribution of boots and gloves, with compensatory proximal hyperhidrosis.^26^^,^^27^ Severe and diffuse loss of this function may lead to thermoregulation and hyperthermia disorders.

Of the available methods, Thermoregulatory Sweat Testing (TST) and Quantitative Sudomotor Axon Reflex Testing (QSART) can be performed.

TST is carried out in a room where it is possible to control temperature and humidity. The patient lies supine on a stretcher, their temperature is monitored by 2 sensors (one for the skin and another one for the oral cavity) and have their body covered by a compound that changes color when the local pH changes by sweat.

The room temperature is increased to 45–50ºC, maintaining a relative humidity of about 35–40%. Skin temperature is maintained between 38.5 and 39.5 ºC and the oral temperature must rise by 1 ºC from the baseline value or reach 38 ºC (whichever is greater). Observation should take place between 30 and 65 minutes. The color change of the reagent on the patient's body indicates the local production of sweat. Digital photographs are taken and an anatomical map of the sweat density is generated, which is then interpreted.

The main limitations of the method are the low availability and inability to distinguish pre- or post-ganglionic impairment. Thus, combining it with a test that is directed at post-ganglionic fibers can help to make this distinction.^31^^,^^34^

QSART evaluates the function of post-ganglionic autonomic fibers related to sudomotor function. It evaluates the production of sweat through the cholinergic stimulus by iontophoresis. Usually, four segments are evaluated — forearm, proximal leg, distal leg and dorsum of the foot; this can provide information about the pattern of involvement: length-dependent or non-length-dependent.

The system consists of a special multi-compartmentalized capsule (a compartment for iontophoretic stimulation, another to measure humidity and a third one that separates the first two), which is in direct contact with the skin, a continuous flow system of dry nitrogen, which passes through the capsule at constant temperature and goes to a hygrometer, which records humidity fluctuation due to local sweat production.

Humidity variation is recorded in a chart on a computer attached to the system. The chart is analyzed mainly for its latency and the area under the curve, with standard values for men and women. The limitations of this method are the difficulty of access and the impossibility of evaluating pre-ganglion fibers.^26^

## Magnetic Resonance Imaging of the Brain and MIBG Scintigraphy

Magnetic resonance imaging can be useful in the diagnosis of multisystem atrophy (MSA) by identifying specific structural changes in the brain, focused on identifying gray matter atrophy patterns.^35^^,^^36^

T1- and T2-weighted images viewed by experienced neuroradiologists have identified classical signs, such as the hot cross bun sign (which represents degeneration of the pons fibers and pontocerebellar fibers, with preservation of the corticospinal tract). This sign appears as a hyperintense cross on the pons, with high specificity (97%), although with low sensitivity (50%). Another observation is the hyperintense signal at the putamen edge with high specificity (90%), although also with less sensitivity (72%).^35^^,^^36^

Over the past few years, there has been significant progress in neuroimaging techniques, using new connectivity and functional techniques, which can improve diagnostic accuracy and determine new markers of disease progression. A multimodal approach with innovative technologies, as part of the diagnostic arsenal, will allow future progress in the diagnosis and research of multisystemic atrophy (MSA).^35^^,^^36^

Regarding Parkinson's disease, the morphological analysis of the midbrain by magnetic resonance imaging, particularly of the substantia nigra and the basal nuclei, presents findings that support the diagnosis of parkinsonian syndromes.^36^

A new and exciting area in MRI is the analysis of brain inflammation. In patients with chronic fatigue syndrome, brain inflammation has been investigated with spectroscopy, measuring levels of various metabolites related to neuroinflammation, including compounds containing choline, myo-inositol, lactate and N-acetylaspartate.^37^ A study evaluating magnetic resonance spectroscopy applied to the entire brain area demonstrated abnormalities of metabolites and temperature distributed throughout the brain, rather than regionally limited.^37^

This finding suggests that chronic fatigue syndrome is a diffuse pathological process that affects the entire brain, which is consistent with the heterogeneous clinical symptoms of the syndrome. These findings, according to the authors, support the hypothesis that the chronic fatigue syndrome is the result of low intensity chronic neuroinflammation.

Another interesting aspect of magnetic resonance imaging is the analysis of cognitive disorders in alpha-synucleinopathies.^38^ Many of these patients have orthostatic hypotension, which leads to transient cerebral hypoperfusion. A suggested hypothesis is that transient or repetitive cerebral hypoperfusion may be responsible for cognitive deficits in these patients.

Structural magnetic resonance imaging demonstrates hyperintensity of the white matter, which may contribute to cognitive defects. There is evidence that orthostatic hypotension is associated with white matter hyperintensity in α-synucleinopathies, partially explaining the relationship between orthostatic hypotension and cognitive impairment.

New applications of functional magnetic resonance imaging show that physiological fluctuations in the white matter observed on the resonance precede the structural changes in white matter hyperintensity, therefore they are more sensitive measures to assess brain impairment.^38^

MIBG (metaiodobenzylguanidine) scintigraphy images can be used to directly quantify cardiac sympathetic innervation in various pathologies, including cardiovascular autonomic neuropathies. Innervation asymmetry may be responsible for predisposition to arrhythmias and sudden death. It can also be used to assess sympathetic reinnervation after adequate treatment.^39^

## Laboratory tests

The most important plasma catecholamines in humans are epinephrine and norepinephrine, both reflecting sympathetic activity. Norepinephrine is released in neuronal sympathetic terminals, and a small portion only reaches systemic circulation. Epinephrine, in turn, is released by sympathetic pre-ganglionic stimulation of the adrenal medulla. Plasma epinephrine and norepinephrine respond differently to stressors. While norepinephrine responds more to cold stimuli, epinephrine is more responsive to hypoglycemia and hypotension.^40^

Standing after resting in a lying position, or tilting the patient on the tilt test, results in blood accumulation in the lower limbs, resulting in a drop in cardiac output. Reflex activation of the sympathetic nervous system results, among other actions, in increased release of norepinephrine by terminals of the sympathetic nerves, reflecting an increase of up to 100% in the plasma circulation of norepinephrine in 5 minutes.

Patients with autonomic failure secondary to dysfunction of the sympathetic post-ganglionic neurons may have reduced concentrations of norepinephrine in the supine position. On the other hand, individuals with autonomic failure for any reason often fail to raise their plasma norepinephrine levels when standing or leaning on the tilt test.

This is due to reduced or no triggers of sympathetic efferents in response to orthostatic stimulus. A subnormal increase in norepinephrine on orthostatic stress is a very specific, although not very sensitive, factor of sympathetic response attenuated by baroreflex-sympathoneural failure or sympathetic denervation.^40^

On the other hand, in patients with hyperadrenergic autonomic dysfunction (such as some patients with mitral valve prolapse or some subtypes of postural orthostatic tachycardia syndrome — POTS — there may be an exaggerated supranormal increase in norepinephrine when subjected to orthostatic stress (standing or when tilting).

In neurogenic orthostatic hypotension, caused by autonomic disorders including cardiovascular autonomic neuropathy (CAN), orthostatic increase in norepinephrine is attenuated. Therefore, an increase in plasma norepinephrine smaller than 60% after 5 minutes of orthostasis supports diagnosis of neurogenic orthostatic hypotension.^40^

Other specific laboratory tests may be requested to investigate various etiologies that may potentially cause dysautonomia, depending on the symptoms and clinical suspicion. Pathologies such as diabetes, amyloidosis, renal failure, autoimmune diseases, neoplasms, mainly of the lung, may require specialized investigation.

**Peripheral Neuropathy — Warning Sign**

The presence of symptoms or diagnosis of peripheral neuropathy may represent a warning sign for Disautonomia investigation. In diabetic patients, more than 50% will have cardiovascular autonomic neuropathy (CAN) when diagnosed with peripheral neuropathy, while almost 100% of patients with CAN will have peripheral neuropathy.

## Treatment

Treatment of dysautonomia and particularly orthostatic hypotension (OH), its main clinical symptom in most cases, must follow a progressive approach that involves both non-pharmacological treatment and use of drugs (Figures 3 and 4).^41^^–^^44^

**Figure 3:** Flowchart on the management of orthostatic hypotension and its differential diagnoses. Divided into groups with increased heart rate in orthostasis, usually observed in OH due to hypovolemia or medication and without increase, as seen in neurogenic hypotension, excluding the use of bradycardic drugs or patients with sinus node disease.

**Figure 4:** Therapeutic approach to dysautonomia.

Source: Author.

The goal of treating patients with OH is to improve debilitating clinical symptoms (specially to reduce the risk of falls) and quality of life, increasing tolerance to longer periods of orthostasis and physical capacity. Having normal blood pressure levels is hardly attainable.

The need for treatment must be based on an individualized analysis of the cases, taking as a reference the severity of the condition and the comorbidities involved. In most cases, particularly in elderly and/or dysautonomic patients, better control of symptoms and vital signs in orthostasis should be sought to help optimize the established therapy.

There is a lack of studies on the treatment of OH, and the existing recommendations are mainly based on small studies. A potential limitation of these studies is that they have not been validated by randomized studies, with a more significant number of patients. Besides, they have the limitation of reflecting mainly the result of acute treatment of OH and, in general, in a heterogeneous group of patients, an aspect that is fundamental since OH involves a range of pathologies that are different to each other in terms of presentation and clinical course.^42^^–^^44^

A consensus of experts in neurogenic OH (NOH) has proposed a stepwise treatment based on 4 steps:

1. evaluating and adjusting pre-existing medications
2. non-pharmacological treatment
3. implementing monotherapy
4. Trying to combine drugs.

According to these authors, there is a recommendation that for each proposed treatment stage there should be a minimum period of 2 weeks of observation to define the symptomatic benefit before migrating to another strategy.^45^^,^^46^

Those involved in the treatment of patients with dysautonomia should always remember that educating the patient, family and caregivers about the mechanisms involved in the genesis of OH and the situations of daily activity that can lead to a drop in blood pressure are the cornerstones of clinical treatment. Staying in hot environments, having hot showers, type and intensity of physical effort, prolonged or quickly achieved orthostatic position, drinking alcohol or having large meals, particularly with carbohydrates, can precipitate or worsen the symptoms.

## Non-pharmacological treatment

### Analysis of the medications in use

Regardless of the etiology of dysautonomia, whenever possible, discontinuation or dose adjustment of drugs that may worsen OH should be considered.^42^^,^^43^^,^^47^ A substantial number of these agents are drugs regularly used by cardiologists.

As the medication is adjusted, it is important to monitor the symptoms of NOH continuously. Some studies recommend using questionnaires created for this purpose.^43^^,^^44^^,^^47^ In cases with a defined indication, antihypertensive drugs with a shorter half-life, preferably with a single nocturnal intake, must be chosen.

Medicines such as nitrates and diuretics, which decrease the preload, must be discontinued or avoided. Other drugs that also worsen or contribute to OH include dopaminergic drugs, anticholinergics, tricyclic antidepressants, α1-blockers (e.g.: tamsulosin) and other antihypertensives.

### Non-pharmacological measures

The next step in treatment is the incorporation of a range of non-pharmacological measures in the patient's daily routine, all with the objective of minimizing the symptoms resulting from NOH. From a practical point of view, these measures are incorporated while a careful review of the pharmacological treatment previously in use is conducted.

For patients with syncope, pre-syncope or recurrent falls, postural instability resulting from OH must be eliminated with greater urgency, and patients must be guided on maneuvers that may reduce venous retention in the lower limbs and digestive tract.^48^

Non-pharmacological measures can be used individually, but are most effective when used in combination or during concomitant titration of pharmacological treatments. Although they are cost-effective and can be combined with pharmacological interventions, non-pharmacological instructions may have low compliance by patients.

### Increased circulatory volume

Patients with NOH need interventions to normalize or expand blood volume. Many of these patients, especially the elderly, present volume decrease secondary to inadequate intake of oral fluid. This may be due to a voluntary restriction of fluid intake, to avoid common conditions such as urinary urgency in the elderly or in patients with neurological diseases.^49^

Adjusting the volume of fluid intake should also consider the geographical area and climatic fluctuations. Water intake is considered a first-choice “drug” in the treatment of NOH.^49^^–^^53^

Also, in acute situations (e.g.: syncope or very symptomatic OH), or when long periods of orthostasis or exposure to heat are expected, rapid water intake is recommended, preferably cold water (500 ml in 2–3 minutes), due to its action in promoting increased sympathetic tone and consequent BP increase.^53^^–^^56^

This acute pressure response starts 5–10 minutes after drinking water, peaking at 20–40 minutes, that is, producing an effect that mimics the use of drugs with a fast short-term effect. The effect of this rapid water intake is due to the hypo-osmolar reflex in the portal circulation and can last for up to 1 hour, enabling an improvement in the symptoms of NOH. The intake of other liquids is ineffective in generating a significant pressure response. Proper hydration can produce acute and chronic effects, with a beneficial clinical impact on patients with NOH.^51^^–^^56^

### Sodium intake

Another important non-pharmacological treatment is monitoring and adjusting the daily supply of salt. Because sodium is considered a negative component of the diet, many patients eliminate or significantly reduce the salt content in the diet, which makes orthostatic symptoms get worse.

For patients with NOH, an intake of 2 to 3 servings of sodium a day (5 to 7.5 g of salt) is recommended. Some cases may require larger intakes, reaching 10 g of sodium. Patients at risk for heart failure, supine hypertension or peripheral edema must be monitored closely as symptoms may get worse and may require adjustments or lower intakes. Excessive salt deprivation should be avoided.^57^^,^^58^

### Diet

In patients with OH, sympathetic activation is not able to compensate for the accumulation of blood in the splanchnic circulation after a meal. In NOH, vasoconstrictive sympathetic activity is deficient and many patients have significant hypotension after food intake.

In individuals with postprandial hypotension, smaller and more frequent meals are recommended.^59^^,^^-^^60^ This type of diet has been shown to be effective in reducing orthostatic symptoms in patients with pure autonomic failure and multisystemic atrophy. There is evidence that a low glycemic diet may have a beneficial effect on OH symptoms. Postprandial hypotension can also be reduced with the caffeine or acarbose.^61^

Anemia leads to decreased blood viscosity and oxygen-carrying capacity with a potential increase in OH symptoms and, therefore, must be prevented and treated.^62^ Vitamin B12 deficit may be associated with postural instability and cause OH, being a reversible cause of some polyneuropathies.^63^ Therefore, changes in diet, as well as supplementation with vitamins and iron in patients with deficiencies of these minerals can be useful in patients with NOH.

### Physical maneuvers to raise blood pressure

OH patients should be informed about simple measures that can be used to increase BP during daily activities. These physical counter-maneuvers include: crossing the legs, squatting and tightening leg, arm, abdomen, bottom or whole-body muscles.^64^ These maneuvers increase cardiac preload, with consequent increases in cardiac output, blood pressure and cerebral perfusion.^64^

The most basic maneuver is activation of the calf muscle pump (“anti-gravity” muscles). If venous valves are competent, muscle activation increases cardiac venous pressure and cardiac filling pressure. Even small BP increases can alter self-regulation and prevent presyncope and syncope.^65^

Patients should be warned that sitting or lying down improves symptoms, but they can recur after returning to orthostatic position. Some evidence points to the beneficial effect of voluntary contraction of the lower limbs for 40 seconds after orthostasis.^47^^,^^48^

It is also useful to train respiratory counter-maneuvers that facilitate venous return of the abdomen and lower limbs to the heart. These respiratory maneuvers use slow deep breathing and inspiratory resistance.

Many patients, particularly those with more severe dysautonomia, need help from others to perform physical counter-maneuvers. They should be advised to get up slowly (over 15 seconds or more), as it has been shown to mitigate blood pressure drop.

### Physical activity

Physical activity and exercise should be encouraged to avoid poor fitness, which is known to worsen orthostatic intolerance.^66^ The mechanisms underlying exacerbation are related to hypovolemia and left ventricular remodeling, leading to deteriorated left ventricular chamber performance. These cardiac abnormalities are reversed by physical training and physical fitness.^67^^–^^69^

However, physical exercise, particularly in cases of OH due to dysautonomy, demonstrated that orthostatic posture, immediately after exercising in the supine position, may exacerbate OH in these patients. This observation is not reproducible in healthy individuals.^67^^–^^69^

As a result, especially in elderly patients with NOH, physical exercise must be supervised by family members or specialized professionals to avoid injuries or falls. In this subgroup of patients, moderate physical training should be prioritized, especially for the lower limbs and physical exercises that do not generate greater gravitational stress, such as cycling in the supine position or water exercises.

Patients should avoid strenuous exercises due to increased body temperature and peripheral vasodilation, with a consequent risk of orthostatic hypotension.^70^^,^^71^

To minimize OH, the patient must be hydrated before and throughout the exercise session and must be warned about the initial risk of OH worsening right after interruption of physical effort.

### Avoiding increased body temperature

Increasing body temperature causes peripheral vasodilation. Patients with NOH should avoid situations that cause an increase in body temperature, such as high-intensity physical exercise, exercise in environments with high temperature and humidity, saunas or hot baths.^72^ Also, as individuals with autonomic failure have an impaired thermal-regulatory capacity, they have higher risk of hyperthermia.

### Head-up tilt sleeping

Head-up tilt sleeping is an important measure. It can be done by sliding a wedge under the mattress or by placing blocks under the legs under the bed head so that the patient's head is 20 to 30 cm higher than the feet, reducing supine hypertension. Smaller tilt angles may not be as successful. Folded pillows placed under the head may not be enough.

Supine hypertension commonly leads to increased nocturia and nocturnal volume depletion. This increase in nocturnal diuresis decreases by raising the bed head. Also, although small, increase in nocturnal gravity stress maintains activation of the renin-angiotensin-aldosterone system, allowing higher pressure in the morning.

The effectiveness of this intervention was questioned in a recent randomized study. However, this study failed to make any distinction between the causes of OH and did not properly monitor hydration and the bed head raising degree, which may have contributed to a negative result. As a result, it should be recommended that at least patients with autonomic failure be told to head-up tilt sleeping. This specific action is not exempt from adverse effects, and may be associated with ankle edema and sliding of the body in bed and consequent feet pain.^73^^–^^75^

### Compressive clothing

Elastic stockings to generate some pressure gradient may be beneficial in the treatment of OH. Compression stockings or bandages reduce the accumulation of peripheral blood in the lower limbs, decrease orthostatic hypotension and reduce symptoms.

Compression must preferably extend to the waist, as most of the stasis occurs in the splanchnic circulation, which contains up to 25% of the resting blood volume. It is necessary to put on the stockings in the morning while lying down, before getting out of bed.

These non-invasive procedures are usually challenging, of low acceptability and require help from third parties, especially in elderly patients and those with neurological diseases. The long-term benefits of these interventions have not been studied. Some authors suggest that an acceptable alternative, due to the low long-term adhesion of compressive clothing, would be wearing cycling clothing, which can offer satisfactory abdominal compression.

In any case, the association between compression techniques (particularly of the abdomen) and physical counter-maneuvers has been shown to be very effective in patients with neurogenic etiology.

An increase in abdominal pressure of 20–40mmHg promoted by abdominal straps combined with physical counter-maneuvers of lower limb contraction results in a significant increase in pressure response to gravitational stress. Studies evaluating non-pharmacological treatment of dysautonomia have found evidence on the use of compressive clothing.^76^^–^^79^

## Pharmacological Treatment

Addition of pharmacological treatment may be necessary in patients with severe OH when non-pharmacological approaches are insufficient to prevent pre-syncopal or syncopal symptoms (figures 3 and 4). This requires probable patient diagnosis, such as NOH, POTS or chronic fatigue syndrome, to be properly considered.

Previous hypertension or supine hypertension, common in patients with dysautonomia and an underlying cardiovascular disease, should also be considered. The treatment of OH is challenging because of the few therapeutic options. Only midodrine and droxidopa (approved in the USA and Japan) have evidence from randomized clinical trials supporting their use in the treatment of OH. Neither of these two drugs is normally available in Brazil.

There are no comparative studies to guide the initial choice of the drug in NOH. Selection of a medication or another, in many situations, will be related to the clinician's preference and experience and the patient's possibility of having access to the medication. Severity and comorbidities (especially heart or kidney failure) should always be taken into account.

These agents can increase BP and blood volume, which may worsen supine hypertension. As a result, the expected improvement in orthostatic hypotension (and the decreased risk of syncope and falls) must be weighed against the long-term risks of hypertension.

Other treatment challenges are the limited availability of clinical evidence and the lack of comparative effectiveness research studies. Below, we present an overview of the main drugs used in the treatment of OH and the recommendations of use.^79^

### Midodrine

Midodrine was the first drug approved by the U.S. Food and Drug Administration (FDA) for OH. It is a prodrug that is quickly converted into its active metabolite, desglimidodrine. This is a selective α1-adrenergic agonist, with short half-life (peak action at 1 hour) and duration of action of about 3–4 hours. Midodrine has been shown to significantly increase blood pressure in orthostasis, while decreasing symptoms of orthostatic intolerance.

A recent meta-analysis also found that midodrine improves clinical outcomes with minimal significant side effects. The dose usually starts at 2.5 mg, and may get to 10–15 mg per dose, up to 3 times a day. Due to the short half-life, a typical dosing schedule is every 4 hours, starting on waking up. It should not be administered at bedtime and patients should avoid lying down for 4 hours after the last dose of midodrine to avoid worsening of supine hypertension.^79^^–^^81^

Given the short half-life, it can also be used as needed, before specific activities related to symptomatic orthostatic hypotension. Side effects of midodrine include supine hypertension, piloerection, tingling of the scalp, urinary urgency or retention and headache.

Midodrine is contraindicated in cases of severe heart disease, bradycardia, history of angina, closed-angle glaucoma, severe occlusive arterial disease, thyrotoxicosis, pheochromocytoma, severe renal failure, Raynaud's disease and proliferative diabetic retinopathy. Care should also be taken in patients with heart failure and chronic renal failure.

### Fludrocortisone

In patients without hypertension or heart failure, fludrocortisone is included in the treatment based on expert opinion and it is the most widely used, especially in countries that do not have the other recommended drugs. Fludrocortisone is a synthetic mineralocorticoid that increases intravascular volume and renal sodium reabsorption. The long-term effects of fludrocortisone on BP, however, are attributed to the increased sensitivity of blood vessels to noradrenaline and angiotensin II. The starting dose is typically 0.05 mg per day and can be increased to 0.3 mg (in a single or divided dose).

Onset of action occurs in 3 to 7 days. Its side effects may include hypokalemia, headaches, peripheral edema, heart failure and supine hypertension. At higher doses, patients may be at increased risk of hypothalamic-pituitary-adrenal axis suppression. 30% of patients stop using the drug due to side effects.

In patients with pre-existing supine hypertension, fludrocortisone is generally not chosen as a first-line medication, with midodrine being the most appropriate one. Formal clinical evidence supporting the use of fludrocortisone for the treatment of neurogenic OH is scarce.^82^^–^^84^

### Droxidopa

The FDA has recently approved droxidopa for the treatment of neurogenic orthostatic hypotension in the United States, especially in Parkinson's disease, multisystemic atrophy and pure autonomic failure. Droxidopa is a synthetic prodrug that is converted into norepinephrine in the brain and peripheral tissues. Circulating levels of norepinephrine increase in 6 hours after droxidopa. The drug has a plasm peak between 1-4 hours, with a average of 2 hours in healthy individuals.

Droxidopa is well tolerated and improves orthostatic tolerance in NOH controlled trials (100–600 mg VO, 3 times a day). Similar to midodrine, droxidopa should not be taken within 5 hours before bedtime. Caution is recommended in patients with congestive heart failure and chronic renal failure. Its side effects include headache, dizziness, nausea and fatigue.^85^

### Other medications

Other drugs include pseudoephedrine, atomoxetine (norepinephrine reuptake inhibitor), yohimbine (α2-adrenergic receptor antagonist), octreotide (somatostatin analogue), ergotamine, erythropoietin and pyridostigmine (cholinesterase inhibitor).^86^^–^^89^

Atomoxetine is a norepinephrine transporter inhibitor approved for the treatment of attention deficit hyperactivity disorder (ADHD). However, in patients with autonomic impairment who have intact peripheral noradrenergic function, this medication can cause a powerful peripheral vasoconstriction, leading to blood pressure increase. This medication is little effective in pure autonomic failure (PAF) due to peripheral impairment of the noradrenergic system.

Pyridostigmine, an acetylcholinesterase inhibitor that increases availability of acetylcholine in nerve endings is supposed to prevent OH. However, it does so by increasing sympathetic nerve activity in response to orthostatic stress, causing changes in baroreceptor sensitivity. It is probably more useful in less severe patients with residual sympathetic function and has the advantage of not worsening supine hypertension. A study has found it is less efficient than fludrocortisone in the OH of Parkinson's disease, but causes less supine hypertension, although this increase in peripheral supine hypertension is not accompanied by a similar increase in central blood pressure with fludrocortisone.^90^^,^^91^

Acarbose, an agent that prevents glucose absorption in the small intestine, decreases the release of gastrointestinal hormones and delays gastric emptying when administered 20 minutes before a meal. This pattern has been shown to be efficient in those cases of postprandial hypotension. It is contraindicated for patients with diabetic ketoacidosis, cirrhosis, inflammatory bowel disease, ulcerative colitis, intestinal obstruction or any chronic intestinal disease that may disrupt digestion or absorption.

Caffeine (200–250 mg or 1 cup of 200 ml coffee per day) in patients who are not chronic users may help by inhibiting peripheral vasodilation. Therefore, it may increase blood pressure in orthostasis.

A recent study showed that dihydroergotamine, in combination with caffeine, can be used as an alternative treatment in patients with autonomic failure and without underlying vascular coronary artery disease.^87^

### Combined pharmacological treatment

There is little data to determine the effectiveness and safety of different combinations of therapy compared to monotherapy for OH. It is recommended to seek the maximum tolerable dose of a single agent and then, if no symptomatic benefit is obtained, consider switching to a different therapy or adding a second agent and titrate from the lowest effective dose.

The most common association in refractory cases is between midodrine and fludrocortisone. The use of water, salt and the preventive measures discussed can also be effective when combined with drugs. A combination with other drugs is possible, always paying attention to dose flexibility (particularly the dose of drugs with short half-life such as midodrine) and strict control of adherence to non-pharmacological treatment.^92^^,^^93^

## Peculiarities of the management of supine hypertension and postprandial hypotension

### Supine and nocturnal hypertension

In patients with OH, especially NOH, we commonly observe an association with supine and nocturnal hypertension, with the severity of nocturnal hypertension correlating with the magnitude of OH. Supine hypertension is distinct from essential hypertension, since most patients are normotensive while sitting and can be severely hypotensive while standing. Approximately 50% of patients with PAF and MSA have supine hypertension. Evaluation of supine and nocturnal hypertension should be performed routinely in patients with NOH, as its presence is a limiter for therapeutic options due to the possibility of adverse effects. ABPM can also be used for diagnostic evaluation and clinical follow-up.

In most patients, there are strong reasons to prioritize the treatment of NOH over supine hypertension. Symptomatic OH carries a variety of posture-related symptoms including dizziness, pre-syncope or syncope, fatigue, cervical spine pain, weakness and visual impairment on orthostasis. All symptoms that can contribute to increased occurrence of falls must be well evaluated, as falls represent some of the most common causes of hospitalization, and it leads to high morbidity and mortality.

To prevent and treat supine hypertension, one should:

1. head-up tilt sleeping;
2. Have carbohydrate-rich meals just before going to bed;
3. Avoid liquids before going to bed;
4. Avoid supine position during the day, especially those patients wearing compression garments or vasopressor drugs.

There are no drugs approved for the treatment of supine hypertension, but there are several potentially useful agents.

In patients who still have some sympathetic tone, a central alpha-2 agonist (clonidine) reduces sympathetic flow when administered late in the afternoon, without exacerbating orthostatic hypotension during the day. It is important to avoid the use of long-acting diuretics and antihypertensive drugs, even if they can control supine hypertension.^89^^,^^92^^,^^93^

**Supine hypertension therapy in dysautonomia**

The cut-off point for BP to start antihypertensive therapy has not been defined and treatment decisions must be made individually. However, antihypertensive drugs may be prescribed with caution if nocturnal BP is predominantly ≥160/100 mmHg (table 2) with administration of shortacting drugs.^89^^,^^92^^.^^93^

Supine hypertension is different from essential hypertension, since most patients are normotensive while sitting and can be severely hypotensive while standing.

**Table 2:** Treatment of Supine Hypertension

Drugs*	Mechanism of action	Usual dose
Captopril	Angiotensin-converting enzyme inhibitor	25 mg at night
Clonidine^a^	Central alpha-2 agonist	0.1–0.2mg after a night meal
Hydralazine	Peripheral smooth muscle relaxation	10–25 mg at night
Losartana	Angiotensin II receptor antagonist	50 mg at night
Nitroglycerin (patch)	Vasodilator	0.1 mg/h (patch — remove in the morning)

* Short-lived antihypertensive drugs should preferably be used to treat supine hypertension. Administration should only be done at night. Remember that many of these medications are usually taken 2–3 times a day and, if taken inadvertently this way, or while awake, they may worsen the symptoms of NOH

a Use of clonidine increases the risk of morning hypotension.

## Postprandial hypotension

Postprandial hypotension (PPH) is commonly observed in patients with OH, but it can occur in isolation, particularly in institutionalized elderly patients. The mechanisms leading to blood pressure drops are not clear. Postprandial hypotension and its extension are favored by glucose intake.

Treatment strategies include: small, frequent meals with low carbohydrate content; drinking water before and during the meal (it is recommended to take 400–500 ml of cold water 30 minutes before meals); minimizing or preferably avoiding alcohol intake; eliminating iatrogenic causes (administration of antihypertensive drugs between meals and not during meals) and caffeine (200–250 mg or 200 ml of coffee), and acarbose (100 mg).
